# Potent acyl-CoA synthetase 10 inhibitors kill *Plasmodium falciparum* by disrupting triglyceride formation

**DOI:** 10.1038/s41467-023-36921-2

**Published:** 2023-03-16

**Authors:** Selina Bopp, Charisse Flerida A. Pasaje, Robert L. Summers, Pamela Magistrado-Coxen, Kyra A. Schindler, Victoriano Corpas-Lopez, Tomas Yeo, Sachel Mok, Sumanta Dey, Sebastian Smick, Armiyaw S. Nasamu, Allison R. Demas, Rachel Milne, Natalie Wiedemar, Victoria Corey, Maria De Gracia Gomez-Lorenzo, Virginia Franco, Angela M. Early, Amanda K. Lukens, Danny Milner, Jeremy Furtado, Francisco-Javier Gamo, Elizabeth A. Winzeler, Sarah K. Volkman, Maëlle Duffey, Benoît Laleu, David A. Fidock, Susan Wyllie, Jacquin C. Niles, Dyann F. Wirth

**Affiliations:** 1 Department of Immunology and Infectious Diseases, Harvard T.H. Chan School of Public Health, Boston, MA USA; 2 Infectious Disease and Microbiome Program, The Broad Institute, Cambridge, MA USA; 3 Department of Biological Engineering, Massachusetts Institute of Technology, Cambridge, MA USA; 4 Department of Microbiology and Immunology, Columbia University Irving Medical Center, New York, NY USA; 5 Wellcome Centre for Anti-Infectives Research, School of Life Sciences, University of Dundee, Dundee, DD1 5EH UK; 6 Center for Malaria Therapeutics and Antimicrobial Resistance, Division of Infectious Diseases, Department of Medicine, Columbia University Irving Medical Center, New York, NY USA; 7 Department of Pediatrics, University of California, San Diego, School of Medicine, La Jolla, CA USA; 8 Tres Cantos Medicines Research and Development Campus, Diseases of the Developing World, GlaxoSmithKline, Tres Cantos, Madrid, Spain; 9 Department of Nutrition, Harvard T.H. Chan School of Public Health, Boston, MA USA; 10 College of Natural, Behavioral, and Health Sciences, Simmons University, Boston, MA USA; 11 Medicines for Malaria Venture, Geneva, Switzerland

**Keywords:** Parasite biology, Pathogens, Malaria

## Abstract

Identifying how small molecules act to kill malaria parasites can lead to new “chemically validated” targets. By pressuring *Plasmodium falciparum* asexual blood stage parasites with three novel structurally-unrelated antimalarial compounds (MMV665924, MMV019719 and MMV897615), and performing whole-genome sequence analysis on resistant parasite lines, we identify multiple mutations in the *P. falciparum* acyl-CoA synthetase (ACS) genes *Pf*ACS10 (PF3D7_0525100, M300I, A268D/V, F427L) and *Pf*ACS11 (PF3D7_1238800, F387V, D648Y, and E668K). Allelic replacement and thermal proteome profiling validates *Pf*ACS10 as a target of these compounds. We demonstrate that this protein is essential for parasite growth by conditional knockdown and observe increased compound susceptibility upon reduced expression. Inhibition of *Pf*ACS10 leads to a reduction in triacylglycerols and a buildup of its lipid precursors, providing key insights into its function. Analysis of the *Pf*ACS11 gene and its mutations point to a role in mediating resistance via decreased protein stability.

## Abstract (web summary)

Drug resistance to current antimalarials is rising and new drugs and targets are urgently needed. Here the authors identify Plasmodium falciparum acyl-CoA synthetase 10 as a new target whose inhibition leads to a decrease in triacylglycerols.

## Introduction

Despite remarkable progress towards malaria elimination, the 2022 World Malaria Report estimated 247 million new cases and 619,000 deaths attributable to malaria in 2021^1^. A limited number of antimalarial drug classes are used to treat the disease, and a degree of resistance to nearly every therapeutic agent has occurred in at least some *Plasmodium falciparum* parasite populations in endemic areas. The identification of new antimalarials that target novel pathways is essential to increase the repertoire of available drugs.

Over the last decade, more than 5 million compounds have been screened against *P. falciparum* in phenotypic screens and over 25,000 hits with low or sub-micromolar activity have been identified^2–7^. Identifying chemically validated targets of these hit compounds can catalyze the use of powerful approaches such as structure-guided drug discovery, fragment screening, or DNA-encoded libraries to accelerate malaria drug discovery and development. The Malaria Drug Accelerator consortium (MalDA) seeks to identify novel drug targets in *P. falciparum* via a chemogenomic approach^8^, focusing on small molecules identified as hits in whole-cell phenotypic screening. A major strategy of this consortium is to apply in vitro evolution of resistant parasites and high-throughput next-generation sequencing, which has identified multiple novel targets and resistance mechanisms^9–12^.

Here we leverage earlier reports of in vitro resistance evolution experiments with three distinct chemical scaffolds (MMV665924, MMV019719 and MMV897615) to gain new insights into the candidate drug targets *Pf*ACS10 and *Pf*ACS11, conserved members of the *P. falciparum* acyl-CoA synthetase (*Pf*ACS) enzyme family^8,12,13^. *Pf*ACS enzymes are most similar to long-chain fatty acid synthetases (ACSLs), which activate free fatty acids (FA) of a preferred acyl-chain of 12–20 by coupling them to coenzyme A in a two-step, ATP-dependent process, resulting in acyl-CoA^14^. Eukaryotic ACSLs show preferences for specific FA substrate lengths and degrees of saturation^15–18^. Parasites scavenge FAs from the host^19,20^ and activation of FAs by *Pf*ACSs allows them to be incorporated into various lipid species essential for parasite growth. This includes accumulation of neutral lipids (triacylglycerols and diacylglycerols) in lipid droplets^21,22^.

Using allelic replacement and thermal proteome profiling, we herein provide evidence of *Pf*ACS10 as being an essential protein and the target of MMV665924, MMV019719, and MMV897615. Inhibition of *Pf*ACS10 leads to a reduction in triacylglycerols and a buildup of its lipid precursors. On the other hand, while allelic replacement of mutations in *Pf*ACS11 phenocopies the selected lines, our conditional knockdown data demonstrate that *Pf*ACS11 is not essential for asexual parasite growth, implying that *Pf*ACS11 may be mediating resistance rather than being a direct target.

## Results

### Allelic replacements confirm the role of *Pf*ACS10 and *Pf*ACS11 in reduced sensitivity to MMV019719 and MMV665924

**Fig. 1:**
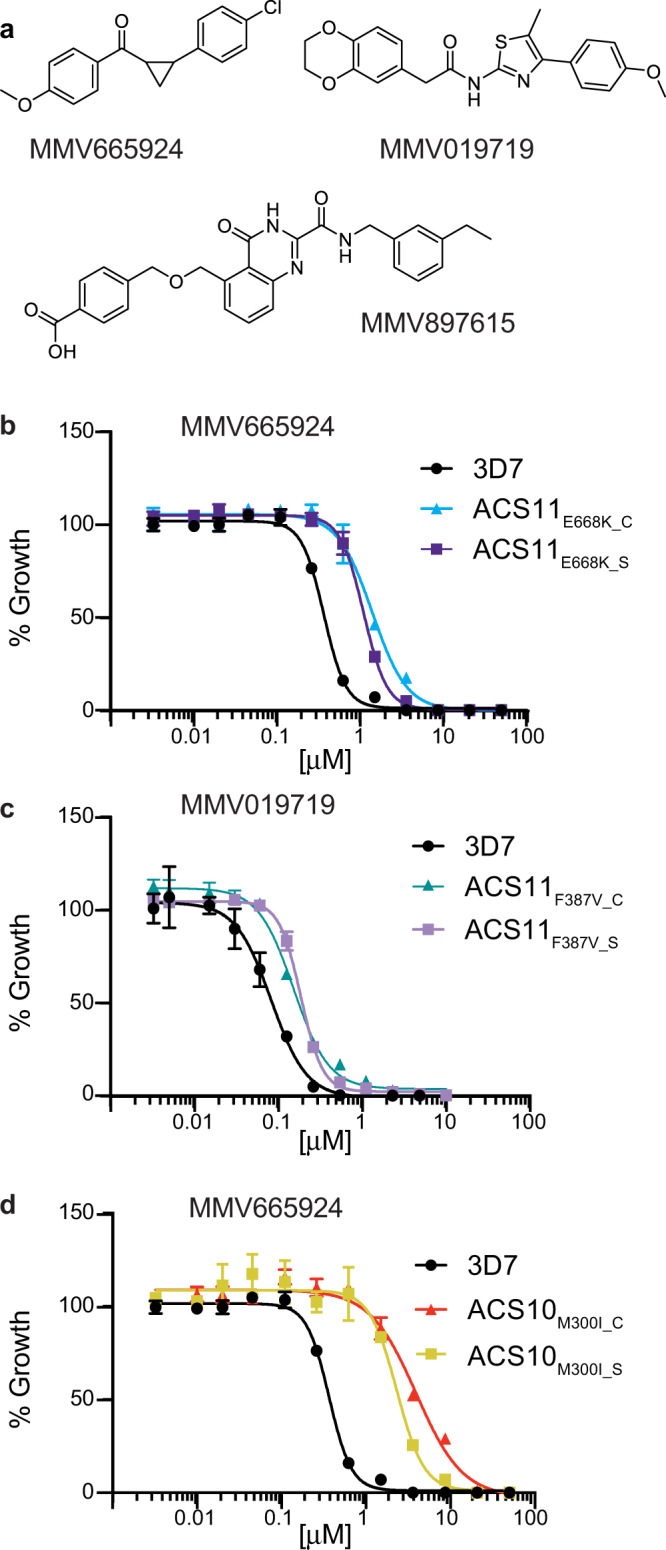
Gene-edited *Pf*ACS10 and *Pf*ACS11 lines phenocopy parasite lines selected to become resistant to a range of antiplasmodial chemotypes. **a** Chemical structures of the compounds used in drug selections. **b**–**d** Dose response curves for a representative example of the parental 3D7 line (black), the selected lines (ACS11_E668K_S_, ACS11_F387V_S_, and ACS10_M300I_S_) and the genetically edited lines (ACS11_E668K_C_, ACS11_F387V_C_, and ACS10_M300I_C_) against the compound used to select for the resistant lines. At least three biological replicates were run for each strain in technical triplicates. Shown are the average ±SD and the non-linear regression curve fit for one biological assay run in triplicate. Statistical significances for EC_50_ are reported in Supplementary Data 1 and source data are provided as a Source Data file.

Previous work by the MalDA consortium identified MMV019719 and MMV665924 as two new phenotypic hits from the chemically diverse Medicines for Malaria Venture (MMV) Malaria Box^8,12^. Resistance selections with MMV665924 resulted in mutations in two new putative targets in the *P. falciparum* acyl-CoA synthetase (*Pf*ACS) enzyme family: *Pf*ACS10 (PF3D7_0525100, M300I) and *Pf*ACS11 (PF3D7_1238800, D648Y, and E668K). Selections with MMV019719 resulted in a single mutation in *Pf*ACS11 (F387V). To verify the role of these mutations in conferring resistance to these two compounds (resistance is defined here as a more than 2-fold shift in EC_50_ values in in vitro culture compared to the parental line), we used CRISPR/Cas9 technology to perform allelic replacements of a subset of the mutations observed (Supplementary Fig. 1 and Supplementary Table 1). Using the 3D7 parental line, we introduced the individual point mutations F387V or E668K into *Pf*ACS11 and M300I into *Pf*ACS10, yielding the gene-edited lines ACS11_F387V_C_, ACS11_E668K_C_, and ACS10_M300I_C_. These parasites phenocopied the increase in EC_50_ values seen in drug-selected compared to wildtype parasites (Fig. 1a–c and Supplementary Data 1). These findings confirm that the mutations in *Pf*ACS10 and *Pf*ACS11 identified by resistance selections with MMV019719 and MMV665924 are sufficient to reduce susceptibility to these compounds. Cross-resistance screening of selected and CRISPR-edited parasite lines revealed that both E668K and F387V in *Pf*ACS11 conferred a similar reduction in susceptibility to parasite lines selected with MMV019719 and MMV665924 (Supplementary Data 1). Compared to the parental line, ACS10_M300I_ parasites also showed a 2.5-fold increase in EC_50_ for MMV019719 (Supplementary Data 1). We did not detect any changes in EC_50_ to other antimalarials (atovaquone, amodiaquine, mefloquine, or quinine) in either the selected or the genetically edited lines compared to the 3D7 parent (One-way ANOVA followed by Dunnett post-test, Supplementary Data 2). These results indicate that *Pf*ACS10 M300I and *Pf*ACS11 E668K and F387V are sufficient to confer resistance to two compounds from distinct chemical scaffolds.

### Mutations in *Pf*ACS10 cause collateral sensitivity

**Fig. 2:**
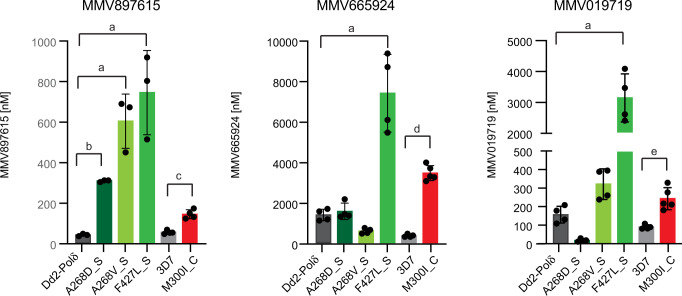
Mutations in *Pf*ACS10 can cause collateral sensitivity to distinct chemotypes. Mean EC_50_ ± SD values for strains containing mutations in *Pf*ACS10 selected in the Dd2-Polδ line (A268D, A268V, and F427L) and in 3D7 (M300I), are shown for MMV897615, MMV665924, and MMV019719. Assays were run in triplicate and at least repeated twice. ANOVA with Dunnett post-tests compared mutations in the Dd2-Polδ line, **a** *p* < 0.001, **b** *p* = 0.005. Two-sided unpaired Student’s *t* test compared 3D7 and ACS10_M300I_C_. **c** *p* = 0.0003, **d** *p* = 0.0000008, **e** *p* = 0.0016. Source data are provided as a Source Data file and in Supplementary Data 1.

More recently, we performed selections in a hypermutator *P. falciparum* line (Dd2-Polδ^23^) with MMV897615, which yielded three novel mutations in *Pf*ACS10 that confer at least a seven-fold increase in resistance (ACS10_A268D_, ACS10_A268V_, and ACS10_F427L_, Fig. 2 and Supplementary Data 1 and 3). MMV897615 (19f) is part of a series of quinazolinone-2-carboxamide derivatives developed to have fast-acting, low nanomolar activity against asexual blood stage parasites, moderate activity against liver stage parasites, and in vivo clearance of *P. falciparum in* a humanized SCID mouse model^24^. We tested whether these mutant lines would also show resistance to MMV665924 and MMV019719 (Fig. 2 and Supplementary Data 1). ACS10_F427L_S_ parasites showed five-fold and 20-fold higher EC_50_ levels for MMV65924 and MMV019719, respectively. Changes at position 268, however, had a more nuanced phenotype. The ACS10_A268D_S_ line showed no shift in susceptibility to MMV665924 but was 10-times more susceptible to MMV019719 compared to the parental line. In contrast, the ACS10_A268V_S_ line was two-fold more susceptible to MMV665924 but two-fold more resistant to MMV019719 compared to the parental line. This differential sensitivity of mutations to different compounds is reminiscent of collateral sensitivity, whereby resistance to one drug increases the susceptibility to another. Overall, our findings on evolved resistance to MMV897615 strengthen the hypothesis that *Pf*ACS10 is a primary target of these compounds.

### A preexisting *Pf*ACS10 mutation in Malawian field isolate confers reduced sensitivity to MMV665924

**Fig. 3:**
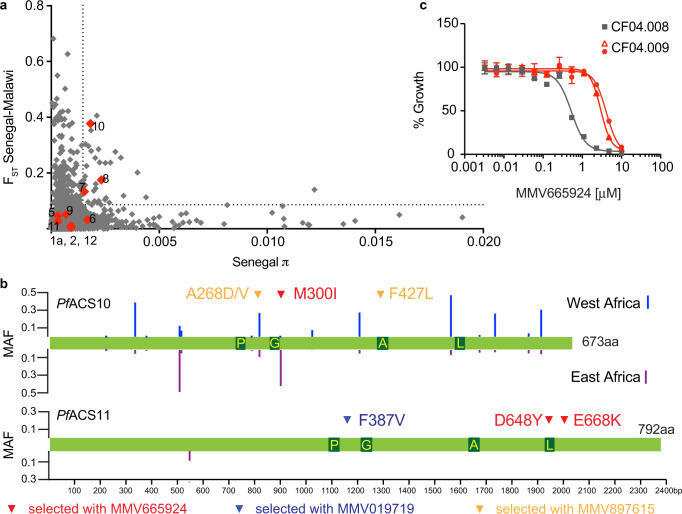
Naturally occurring variants in *Pf*ACS10 can confer resistance to MMV665924. EC_50_ **a** Pairwise nucleotide diversity (π) and divergence (*F*_*ST*_) between Malawian and Senegalese populations for 4614 genes in *P. falciparum*. Members of the ACS family are indicated in red. The 95th percentile is indicated by a dotted line. Overall, pairwise π of Malawi and Senegal is highly correlated (two-tailed Pearson *R*^2^ = 0.94, *p* < 1 × 10^−15^) and generally relatively low (median of 0.0003207 and 0.0003214 for Senegal and Malawi, respectively). **b** Distribution of non-synonymous single nucleotide polymorphism with global MAF > 0.01 from globally diverse isolates (www.malariagen.net/pf3k). The length of the bars represents the local MAF of each variant in West (blue) or East (purple) Africa. The selected variants are indicated by red, blue, and yellow arrows. Dark green boxes indicate the ACS motifs: P: P-loop, G: Gate motif, A: Adenine binding site, L: Linker. **c** Representative dose-response assay for a clonal line of CF04.008 (ACS10 M300, gray) and two clonal lines of CF04.009 (ACS10 M300I, red) from Malawi. Assays were run in triplicate and repeated twice. Shown are the average ±SD and the non-linear regression curve fit for one biological assay run in triplicate. Source data are provided as a Source Data file. MAF minor allele frequency, π pairwise nucleotide diversity within each country, *F*_*ST*_ divergence.

To understand whether there are preexisting variants in the *Pf*ACS10 and *Pf*ACS11 genes in natural populations (Supplementary Data 4), we compared the diversity within and the divergence between parasite cohorts obtained from Malawi and Senegal (Fig. 3a). In both cases, *Pf*ACS10 was in the 95^th^ percentile for pairwise nucleotide diversity (π) within each country as well as divergence (*F*_*ST*_) between Malawi and Senegal.

Among the common natural variants (Fig. 3b), we found a mutation that had also evolved in vitro in cultured parasites selected with MMV665924 (ACS10_M300I_S_). The ACS10^M300I^ variant was present within 78% of Malawi isolates from a high-transmission area^25^. To test whether the M300I variant in its natural genetic background confers resistance to MMV665924, we culture-adapted isolates from two patient-derived samples in Malawi^26^—one containing no mutations in *Pf*ACS10 (CF04.008) and the other harboring the M300I substitution (CF04.009). Inhibitory growth assays of cloned isolates showed over a five-fold higher EC_50_ in the M300I isolate (Fig. 3c and Supplementary Data 1), providing evidence that the M300I variant in its natural genetic background could contribute to reduced parasite susceptibility to MMV665924.

### Resistance-conferring mutations in *Pf*ACS10 and *Pf*ACS11 line the FA binding pocket

**Fig. 4:**
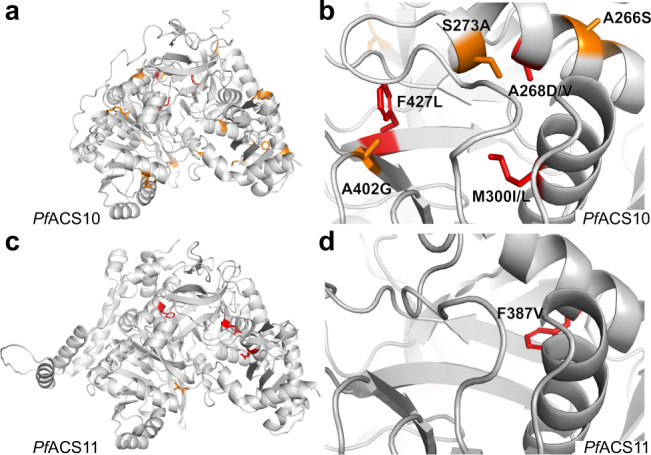
Resistance-conferring mutations and naturally arising variants occur in the predicted fatty acid binding pockets of *Pf*ACS10 and *Pf*ACS11. **a** AlphaFold model of the structure of *Pf*ACS10. **b** Mutations conferring resistance to MMV665924 and MMV897615 occur within the fatty acid binding pocket of *Pf*ACS10. The position of the resistance-conferring mutations M300I, F427L and A268D/V are depicted in red. Several non-synonymous genetic variants in the MalariaGen Pf3k database with high minor allele frequency also occur within the fatty acid binding pocket (A402G, S273A, A266S). **c** AlphaFold model of ACS11. **d** The F387V mutation (depicted in red) is localized to the predicted fatty acid binding pocket of the *Pf*ACS11 protein. Amino acid positions with non-synonymous genetic variants in the MalariaGen Pf3k (www.malariagen.net/pf3k)  database with minor allele frequency >0.01 are depicted in orange (Fig. 3 and Supplementary 4). Resistance-associated mutations are depicted in red.

To examine the distribution of naturally occurring variants (Supplementary Data 4) and resistance-associated mutations in the protein structures of *Pf*ACS10 and *Pf*ACS11, we made use of homology models generated by Alphafold^27^ (https://www.alphafold.ebi.ac.uk/). The mutations in *Pf*ACS10 associated with resistance or collateral sensitivity to MMV019719, MMV665926 and MMV897615 (M300I, A268D/V and F427L) all occur within an internal pocket of the *Pf*ACS protein models (Fig. 4), which in other organisms accommodates the acyl-chain of FA substrates^14,28–30^. Several naturally occurring variants in *Pf*ACS10 (S273A, A402G, A266S) were also found near the resistance-associated mutations in the FA binding pocket.

In *Pf*ACS11 only the F387V mutation lines the FA binding pocket. The D648Y and E668K mutations occur in adjacent folds facing the nucleotide-binding region of the C-terminal domain of *Pf*ACS11. Previously reported *Pf*ACS11 mutations (E660K and K462N), found to confer resistance to pantothenamide bioisosteres, also occur within this region^31,32^. These observations suggest that mutations in the FA binding pocket may confer resistance by disrupting inhibitor binding.

To explore whether mutations in *Pf*ACS10 and *Pf*ACS11 could indeed alter interactions with inhibitors binding the FA pocket, we tested Triacsin C, a polyunsaturated FA mimic that inhibits long-chain ACS enzymes in other organisms, on a subset of parasites carrying individual mutations in *Pf*ACS10 (M300I) or *Pf*ACS11 (F387V, D648Y, and E668K). Mutant parasites showed differential sensitivity to this compound, with stronger effects resulting from the mutations *Pf*ACS10 M300I and *Pf*ACS11 F387V that line the FA pocket. These results are consistent with the hypothesis that these mutations alter interactions within the FA substrate-binding pocket of the enzymes (Supplementary Fig. 2 and Supplementary Data 2).

### *Pf*ACS10 is essential during asexual in vitro growth

**Fig. 5:**
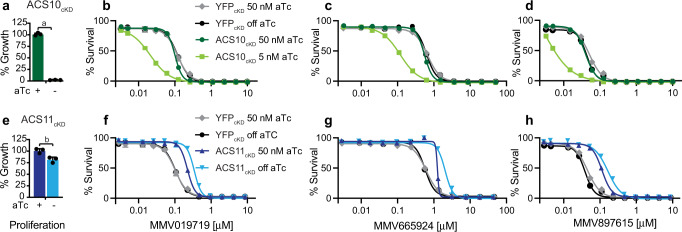
Knockdown of *Pf*ACS10 leads to hypersensitivity to profiled inhibitors. The conditional knockdown line for *Pf*ACS10 was generated with the TetR-aptamer system, similar to the previously generated ACS11cKD and YFP_cKD_ lines^40^. Viability of the lines was tested by removing anhydrotetracycline (aTc) and measuring parasite proliferation by luminescence of the luciferase reporter gene after 72 h. **a** Loss of *Pf*ACS10 resulted in a complete block of growth, whereas **e** loss of *Pf*ACS11 resulted in a 20% growth reduction. Shown is one biological replicate run in triplicate. Tests for significance used a two-sided unpaired Student’s *t* test: a: *p* = 0.0000005, b: *p* = 0.012. Example of dose response curves for **b**, **f** MMV019719, **c**, **g** MMV665924, and **d**, **h** MMV897615, tested in the presence of high aTc (50 nM), or low aTc (5 nM aTc for ACS10_cKD_ to allow for minimal growth), or no aTc for ACS11_cKD_ and YFP_cKD_. ACS10_cKD_ parasites in low aTc were significantly more susceptible to all compounds as compared with high aTc. Shown are the average ±SD and the non-linear regression curve fit for one biological assay run in triplicate. In contrast ACS11_cKD_ parasites were less susceptible to the compounds in the absence of aTc, whereas the aTc concentration had no effect on the YFP_cKD_ line. Statistical significances for EC_50_ are reported in Supplementary Data 1 and source data are provided as a Source Data file. YFP yellow fluorescent protein, aTc anhydrotetracycline.

To further investigate the role of *Pf*ACS10 in the resistance mechanism, we used the TetR-DOZI aptamer system^33^ to generate *Pf*ACS10 conditional knockdown parasites (ACS10_cKD_). Withdrawal of anhydrotetracycline (aTc) results in reduced protein levels of the aptamer-tagged gene, however, we were not able to introduce a protein-tag to *Pf*ACS10 to measure protein reduction under inducible conditions. Nevertheless, removal of aTc was observed to cause loss of parasite proliferation in the ACS10_cKD_ line (Fig. 5a). These results confirm the essentiality of *Pf*ACS10 in asexual blood stage parasites, as previously suggested from a *P. berghei* knockout model^34^. Knockdown of *Pf*ACS10 resulted in significant parasite hypersensitivity to MMV019719, MMV665924 and MMV897615 (Fig. 5b–d, unpaired Student’s *t* test *p* < 0.01, Supplementary Data 1), consistent with an inhibitory interaction of these compounds with *Pf*ACS10 directly or indirectly within the same pathway^35^.

### MMV897615 interacts directly with *Pf*ACS10

To determine whether *Pf*ACS10 is a direct target of the compounds or is otherwise involved in a resistance mechanism, we used thermal proteome profiling (TPP), which is an effective and unbiased method to demonstrate compound-target engagement. This approach is based on the principle that binding of a drug to its protein target can significantly alter the thermal stability of that protein^36^. Here, we employed a whole-cell version of TPP to identify the molecular target(s) of MMV897615, by treating cultured parasites with 500 nM MMV897615 or DMSO vehicle for 1 h prior to cell lysate preparation. We established full melt curves for 2311 proteins over the temperature range of 37 °C to 72 °C, representing >43% coverage of the *P. falciparum* proteome. This compares favorably with the coverage reported for previous TPP and cellular thermal shift assays coupled with mass spectrometry (MS-CETSA) studies with *P. falciparum*^37,38^. TPP datasets were analyzed using the standard melting temperature (*T*_m_)-based method, as described in an earlier TPP publication^39^. This analysis assesses individual protein melt curves using several criteria including curve R^2^, variability in *T*_m_ values within the control sample, maximum curve plateau, and minimum slope. Melting point differences (Δ*T*_m_ = *T*_m, treated_–*T*_m, control_) are then established for every detectable protein. Only the most significantly affected proteins are selected as potential “hits” by applying an FDR-adjusted z-test to Δ*T*_m_ data_._ Proteins with a *p*-value <0.1 in two separate technical replicates are considered “hits”. Hits found in two biological replicas are considered as putative targets.

**Fig. 6:**
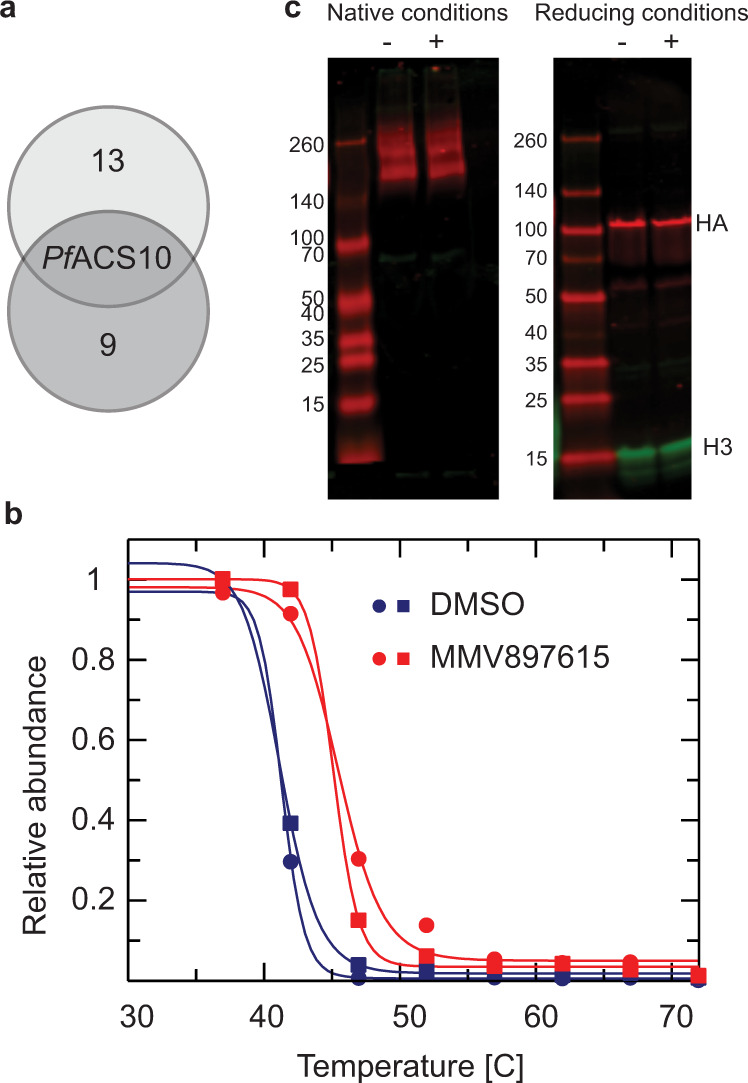
Thermal protein profiling (TPP) confirms *Pf*ACS10 as the primary target of MMV897615. **a** Venn diagram of proteins displaying the most significant thermal shifts in the presence of MMV897615 from duplicate experiments (biological replicates). **b** Melt curve for *Pf*ACS10 following incubation with 500 nM MMV897615 or vehicle (0.1% DMSO) in the two independent experiments. The mean shift in melting temperature (Δ*T*_m_) for *Pf*ACS10 was 4.13 °C. Data from the two independent duplicate experiment are presented in Supplementary Data 5. **c** Western blot showing protein lysate of ACS11_cKD_ parasites cultured in the presence of 500 nM aTc. Protein lysates was either exposed to 10 mM MMV665924 and MMV019719 each (+), or control DMSO (−) for 30 min at room temperature to test for effect of MMV665924 and MMV019719 on protein stability. Lysates were either run on a non-denaturing gel or on a SDS gel, transferred to a membrane, and detected with anti HA antibodies. No effect of MMV665924 and MMV019719 on dimerization could be detected. Source data are provided as a Source Data file. aTc anhydrotetracycline.

Analysis of our TPP datasets identified 14 “hits” in the first biological replicate and 10 in the second (Supplementary Data 5). However, the only protein identified as a putative target in both datasets was *Pf*ACS10 (Fig. 6). Other *Pf*ACS enzymes were successfully identified in our TPP datasets; their thermal stability remained unchanged in the presence of MMV897615. Because the *T*_m_ method represents the most stringent and robust approach to “hit” identification, we have a high degree of confidence that *Pf*ACS10 interacts directly with MMV897615.

### Reduced *Pf*ACS11 level is associated with increased resistance

Previous work has demonstrated that mutations in *Pf*ACS11 have been found to confer resistance to multiple acetyl-CoA synthetase (AcAS) inhibitors^31,32,40^ and that *Pf*ACS11 is not essential for asexual blood stage parasite growth described in a *P. berghei* knockout model^34^ and a *P. falciparum* PiggyBac essentiality screen^41^. Our work reported herein demonstrates that resistance is conferred by the mutations selected by MMV665924 and MMV079179. To further investigate the mechanism of resistance, we tested the susceptibility of the previously described ACS11_cKD_ and control YFP_cKD_ parasites^40^ to MMV665924, MMV079179, and MMV897615 in the presence or absence of aTc. Knockdown of *Pf*ACS11 protein levels resulted in a higher level of resistance to all three drugs (Student’s *t* test *p* < 0.001, Supplementary Data 1 and Fig. 5f–h). Interestingly, even in the presence of 50 nM aTc, the ACS11_cKD_ was 2-fold resistant as compared to the YFP control, perhaps due to the HA-tag resulting in protein destabilization. These results suggest that a reduction or destabilization in *Pf*ACS11 protein might drive the resistance mechanism.

Further evidence for destabilization comes from the analysis of our TPP data set. Non-parametric analysis of response curves (NPARC), an alternative and perhaps less stringent method for hit identification, indicated that *Pf*ACS11 was destabilized in the presence of MMV897615 (Supplementary Data 5). Unlike the *T*_m_ method of hit identification described above, NPARC takes into account the entire melting curve, comparing the goodness of fit of the experimental data to a null model that assumes that the protein is unaffected by drug treatment, or an alternative model that assumes that the protein is affected by the treatment. An FDR-adjusted *p*-value is generated that denotes the significance of the effect of the drug on protein melting behavior. In this study, proteins with an NPARC *p*-value < 0.01 were considered “hits” and those hits common to both biological replicas were considered putative targets. Destabilization of a protein can indicate disruption of a protein complex or dimer. A Western blot with protein lysate, prepared under non-denaturing conditions from an ACS11_cKD_ parasite line expressing HA-tagged *Pf*ACS11, showed a double band between 140 and 260 kDa, whereas a single band at around 100 kDa was present in denaturing conditions (Fig. 6c). This result suggests that *Pf*ACS11 may oligomerize or form a complex with other proteins. To test for whether *Pf*ACS11 forms a complex with other *Pf*ACS proteins or other interacting partners, we performed pull-down experiments with ACS11_cKD_ trophozoites expressing HA-tagged *Pf*ACS11. Results revealed no significant enrichment for any proteins other than *Pf*ACS11 (Supplementary Fig. 3 and Supplementary Data 6). Taken together, these results suggest that *Pf*ACS11 is a potential resistance mediator, as opposed to a direct target, and that resistance is likely mediated via loss of function.

### *Pf*ACS10 shows preference for FAs with an acyl-chain length of 18 carbons

*P. falciparum* parasites can scavenge a broad range of FA substrates from the host during asexual blood stage development^42^. Hence, we tested the effect of *Pf*ACS10 knockdown on total FA composition of infected RBCs using gas chromatography-flame ionization detection (GC-FID). As observed previously^43^, parasite invasion alters the FA composition of the erythrocyte drastically, with 60% of FA made up by just three FA in the parasitized red blood cells: 16% stearic (18:0), 13% palmitic (16:0) and 30% oleic acid (18:1n-9c) (Supplementary Data 7 and 8).

**Fig. 7:**
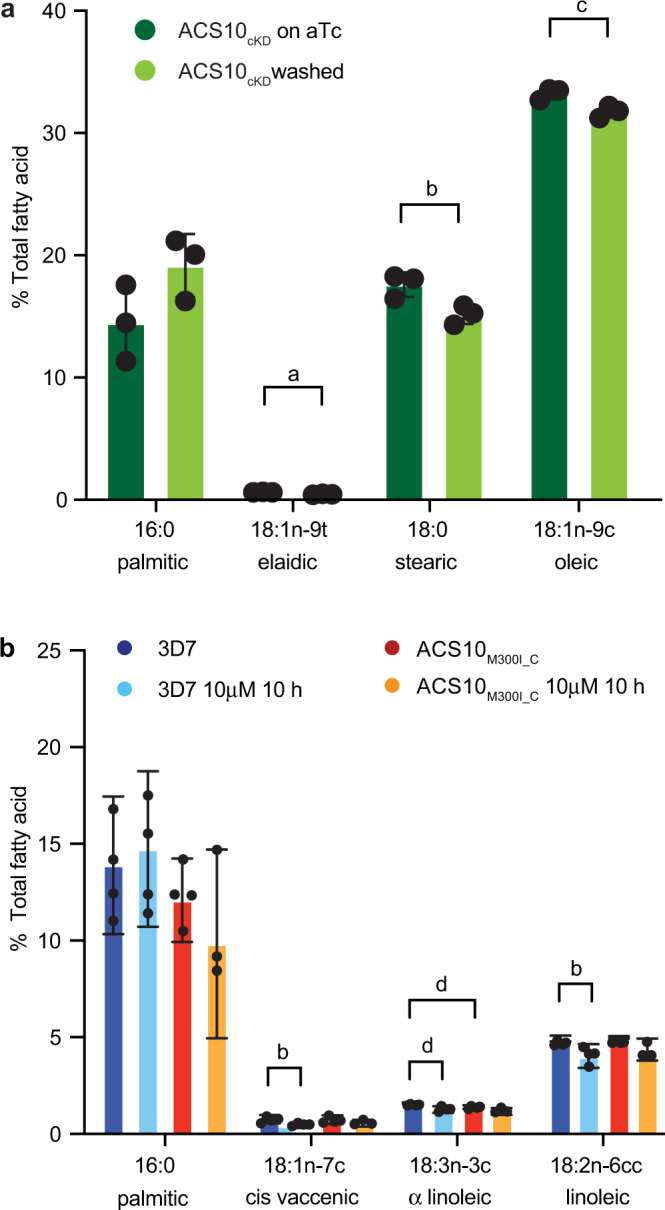
*Pf*ACS10 prefers fatty acid species of 18 carbon length. **a** ACS10_cKD_ parasites were washed 24 h prior to enrichment for trophozoites by MACS purification to allow for reduced levels of *Pf*ACS10. Under these aTc washout conditions, there was no visible defect in the ACS10_cKd_ line. FAs form infected erythrocytes were extracted and submitted to gas chromatography-flame ionization detection (GC-FID); results were normalized to total FA. Shown are the mean ± SD of three biological replicates for relevant fatty acids. **b** 3D7 and ACS10_M300I_C_ were enriched for young trophozoites and exposed to 10 mM MMV665924 or DMSO for 10 h, after which FAs were extracted and analyzed by GC-FID. Shown are the means ± SD of four biological replicates for relevant FAs. Test for significance used two-sided unpaired Student’s *t* test, a: *p* = 0.006, b: *p* = 0.03, c: *p* = 0.02, d: *p* = 0.01. The complete data for the GC-FID are available in Supplementary Data 7 and 8. aTc anhydrotetracycline, FA fatty acid.

After removal of aTc, there were small but significant reductions in stearic, oleic and elaidic (18:1n-9t) acids in ACS10_cKD_ parasites compared to full aTc controls (mean of 15, 5, and 27% reductions respectively, unpaired Student’s *t* test *p* < 0.05, Fig. 7a and Supplementary Data 7). These data suggest that *Pf*ACS10 could be involved in the uptake and retention of FAs with a preferred acyl-chain length of 18.

We next tested whether treatment with one of the compounds directly changes the FA composition in infected erythrocytes. Upon treatment of 3D7 parasites with MMV665924, there was a significant reduction of alpha-linoleic (18:3n-3c), linoleic (18:2n-6cc), and cis-vaccenic (18:1n-7c) acids (20, 18, and 45% reductions respectively, p < 0.05 unpaired Student’s *t* test, Fig. 7b), and a significant (28%) increase in heptadecanoic (17:0) acid (Supplementary Data 8). In contrast, when we treated the resistant ACS10_M300I_C_ line with MMV665924, we observed a 16% increase in arachidonic acid (20:4n-6c) but no significant reduction in any specific FA (*p* < 0.05 unpaired Student’s *t* test, Supplementary Data 8). Alpha-linoleic and rumenic acid (18:2n-7ct) were significantly reduced in the untreated ACS10_M300I_C_ line compared to 3D7 (12 and 46%, respectively, Fig. 7b and Supplementary Data 8). As in the knockdown experiments, FAs with a chain length of 18 carbons were the most affected.

*P. falciparum* parasites can sustain limited growth in “minimal media” that is essentially free of FAs, except for palmitic (16:0) and oleic acid (18:1n-9c)^44,45^. We tested whether supplementation of minimal media with some of the FAs most reduced by MMV665925 could alter parasite survival under MMV665924 treatment. Indeed, addition of linoleic (18:2n-6cc) and cis-vaccinic (18:1n-7c) acid significantly increased the EC_50_ (two-fold) compared to minimal media (Supplementary Fig. 4, *p* < 0.05 by ordinary one-way ANOVA compared to growth in minimal media, followed by Dunnett post-test). Addition of myristic (14:0), palmitoleic (16:1n-7c) or stearic acid (18:0) did not have a significant effect on the EC_50_ of MMV665924. These data suggest that formation of linoleic and cis-vaccinic acids might indeed be inhibited by MMV665924, and an exogenous source can rescue parasite growth.

### Inhibition of *Pf*ACS10 leads to defective conversion of diacylglycerols to triacylglycerols

**Fig. 8:**
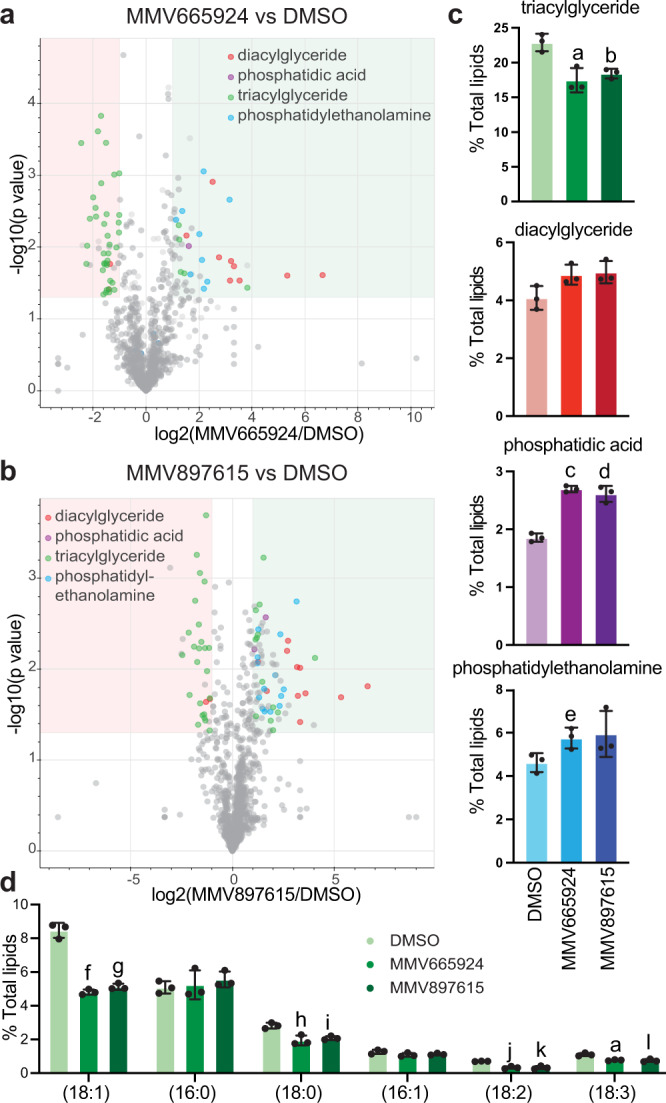
Drug treatment inhibits diacylglycerol to triacylglycerol conversion in trophozoites. Young 3D7 trophozoites were enriched based on their magnetic binding to MACS columns and were treated with DMSO, 10 mM MMV665924 or 1 mM MMV897615 for 8 h. Lipids were extracted and submitted for Liquid Chromatography / Mass Spectrometry (LC/MS). Three biological replicates were performed, and results of each sample were normalized to total lipid composition of the sample. Volcano plots showing lipid species for DMSO-treated parasites vs MMV665924 (**a**) or MMV897615 (**b**), lipid species that are more than 2-fold different and have a p value <0.05 (two-sided unpaired Student’s *t* test) are shaded. **c** Changes in relevant lipid subclasses upon drug treatment compared to DMSO control. **d** The contribution of each individual FA species to the total TAG pool was analyzed and compared between the DMSO control and the drug-treated parasites. Data are shown for the six most frequently detected FAs. Results were obtained from three biological replicates and are shown as means ± SD. Differences were tested using a two-sided unpaired Student’s *t* test. a: *p* = 0.01, b: *p* = 0.006, c: *p* = 0.00009, d: *p* = 0.001, e: *p* = 0.04, f: *p* = 0.0002, g: *p* = 0.0003, h: *p* = 0.004, i: *p* = 0.003, j: *p* = 0.0008, k: *p* = 0.002, l: *p* = 0.008. The complete data are available in Supplementary Data 9 and 11, and in the Source Data file. FA fatty acid, TAG triacylglycerol.

*Plasmodium* parasites modify the lipid composition of their intracellular environment during their asexual lifecycle and especially increase the amount of natural lipids (diacylglycerols (DAGs) and triacylglycerols (TAGs)) in the infected red blood cell^21^. To investigate which lipid species were affected by drug treatment, we used liquid chromatography-mass spectrometry (LC–MS) analysis. We detected a total of 996 lipid species, which could be subdivided into 27 different subclasses (Supplementary Fig. 5 and Supplementary Data 9). Overall, we observed a significant 20–24% reduction in TAGs and a 40–45% increase in phosphatidic acid in both drug-treated parasite samples compared to DMSO mock-treated controls (MMV665924 and MMV897615, respectively, unpaired Student’s *t* test *p* < 0.05, Fig. 8). Treatment with either compound elicited a similar response (unpaired Student’s *t* test *p* < 0.05, Supplementary Fig. 6 and Supplementary Data 10).

TAGs have three FA moieties, and we analyzed the contribution of each individual FA species detected in the total TAG pool. Only six FA species (18:1 16%, 16:0 10%, 18:0 9%, 16:1 6%, 18:2 5% and 18:3 5%) made up over half of the TAG pool. We compared the contribution of these six FAs from treated and untreated parasites (Fig. 8d). Interestingly, all four 18-carbon FA species were significantly less abundant in the treated parasites compared with the DMSO control (unpaired Student’s *t* test *p* < 0.01), whereas the amount of 16-carbon FAs was not affected. These results are consistent with the GC-FID data, where we also observed the biggest reduction in FAs with 18 carbons. These combined data suggest that MMV665924 or MMV897615 treatment inhibits incorporation of 18-carbon FA into newly forming TAGs.

### MMV019719, MMV665924, and MMV897615 act during peak membrane biosynthesis

**Fig. 9:**
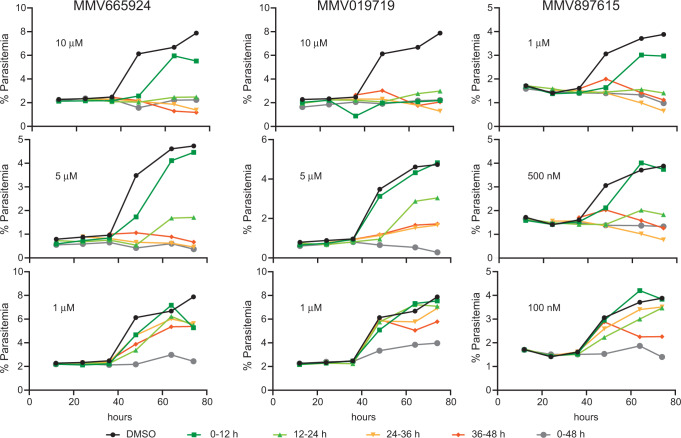
MMV019719, MMV665924, and MMV897615 are most active against late-stage parasites. 0–6 h old parasites were exposed for 12 h windows to three different concentrations of compound (low, intermediate, and high). Smears were taken before addition of the drug (start) and every 12 h after drug exposure. Representative images for the intermediate concentration are shown in Supplementary Fig. 4. The parasitemia was measured by flow cytometry every 12 h and into the second lifecycle to determine viable parasites, as indicated by an increase in parasitemia. Parasitemia can be seen to increase at 48 h in the untreated DMSO control as well as for parasites treated at 0–12 h for MMV665924 and MMV897615 at all concentrations tested and at intermediate and low concentrations for MMV019719. Treatment of intermediate concentrations also resulted in an increase in parasites exposed at 12–24 h. Exposure of high and medium concentrations after 24 h almost completely inhibited growth. 100,000 cells were counted per timepoint and an example of the gating strategy is shown in Supplementary Fig. 8. Source data are provided as a Source Data file.

To further investigate the biological activity of MMV019719, MMV665924 and MMV897615 in asexual blood stage parasites, we determined the life-stage when the compounds were the most potent. We exposed synchronized parasites to each compound for 12 h windows at three different concentrations during an intraerythrocytic cell cycle and measured parasitemia throughout and into the following cycle (Fig. 9 and Supplementary Figs. 7 and 8). All compounds showed the highest activity against trophozoites (24–36 h and 36–48 h post-invasion) and minimal activity against early ring stages (0–12 h). Parasites treated 24 h post-invasion formed abnormal schizonts that did not progress to the next cycle (Supplementary Fig. 7). This observation is consistent with the transcript levels of *Pf*ACS10 and *Pf*ACS11, which peak in the later stages of asexual blood stage development^46–48^ (http://plasmodb.org^49,50^) when the demand of FAs is at its highest.

## Discussion

Here, we demonstrate that mutations in *Pf*ACS10 and *Pf*ACS11, two members of the acyl-CoA synthetase family in *P. falciparum*, cause reduced sensitivity to three chemically distinct and novel compounds (MMV665924, MMV019719, and MMV897615). The mutations in *Pf*ACS10 are predicted to line the FA binding pocket, suggesting that the compounds might interfere with substrate binding. We show that *Pf*ACS10 is essential in asexual blood stage parasites by conditional knockdown and demonstrate that *Pf*ACS10 directly interacts with MMV786519 by TPP. ACS enzymes are responsible for the activation of free FAs needed for a variety of biological processes, and we show that drug treatment leads to a reduced level of TAGs and a buildup in the lipid precursor phosphatidic acid and DAGs. These findings highlight a new vulnerability of the parasite that is amenable to antimalarial drug discovery^21^. The use of ACS inhibitors as therapeutic compounds has been explored in cancer treatment^51^ as well as in parasites such as *Giardia*^52^ and *Cryptosporidium parvum*^53^. More importantly, the *Pf*ACS10 inhibitor MMV1582367 (GSK701) is in a phase I clinical trial for the treatment of uncomplicated malaria (NCT05507970).

In *P. falciparum*, the *Pf*ACS gene family is expanded^13^, when compared to other eukaryotic organisms. The expanded functional repertoire of this expanded family is not fully delineated, but previous work including a PiggyBac transposon screen^41^ points to *Pf*ACS10 as an essential gene. Furthermore, our conditional knockdown studies directly demonstrate this gene’s essentiality for the growth of asexual parasites in culture.

Several lines of evidence point to a role for the *PfACS11* protein in conferring resistance via a loss of function mechanism. Allelic replacements studies demonstrate that the mutations confer resistance and conditional knockdown of *Pf*ACS11 protein by 80% (as previously established by Summers et al.^40^) resulted in resistance to all three compounds. ACS11_cKD_ parasites cultured without aTc displayed a 20% reduction in growth, suggesting that *Pf*ACS11 is not essential in asexual stages in vitro. However, it is possible that a stronger reduction of the protein level is needed to inhibit parasite growth, as has been shown for plasmepsin V^54^. *PfACS11* also plays a role in conferring resistance not only to the compounds reported here but also to pantothenamides^31^ and MMV019721^40^ that target acetyl-CoA synthetase, which activates acetate rather than FAs. Tagging the *Pf*ACS11 locus rendered parasites more resistant to MMV665924, MMV019719 and MMV897615 as well as to MMV019721^40^. It has been shown for other proteins that addition of 3’ aptamers can substantially reduce protein levels, even in the presence of 500 nM aTc^33^. We speculate that loss of *Pf*ACS11 function could confer decreased sensitivity to these compounds. Interestingly, NPARC analysis of TPP detected a decrease in *Pf*ACS11 stability in the presence of MMV897615, which may indicate destabilization of a protein complex through direct interaction with the compound. ACS enzymes often function as dimers^14,55,56^ and Western blot of a native gel suggest that *Pf*ACS11 could indeed form a homodimer (Fig. 6c). We have not found any evidence based on pulldowns of *Pf*ACS11 directly interacting with any of the other twelve *Pf*ACSs, or any other protein (Supplementary Fig. 3, Supplementary Data 6). Overall, our findings suggest that while the compounds can directly interact with both enzymes, the primary growth-inhibitory effect of MMV665924, MMV019719, and MMV897615 is derived from their binding to the FA binding pocket of *Pf*ACS10 rather than from the destabilization of *Pf*ACS11. *Pf*ACS11 is therefore interesting in terms of its biological function but is a less compelling drug target, and further studies are needed to understand its role in resistance.

As resistance-causing mutations appear to line the FA binding pocket of *Pf*ACS10, we propose that the compounds interfere with the ability of *Pf*ACS10 to activate free FAs. ACSs can activate a variety of FAs and are often acyl-chain length-specific^15–18^. Individual ACSL expression has also been correlated with individual acyl-CoA levels in mammalian cells^57^. We have shown that a knockdown of *Pf*ACS10 leads to a reduction in endogenous stearic and oleic acids—some of the most abundant FAs in the parasite—suggesting a preference of *Pf*ACS10 for C18 FA length. Treatment with MMV665924 led to a reduction in linoleic, alpha-linoleic, and cis vaccenic acid, which can be directly derived by desaturation from stearic and oleic acid upon activation by an ACS. Discrepancies in the specific species of C18 FA changing between knockdown and drug treatment could reflect different timing and duration of the perturbations. It is also possible that *Pf*ACS10 disruption could alter subcellular trafficking of FAs, which could not be assessed here. Further functional studies of *Pf*ACS10 are needed to confirm the substrate specificity and function of *Pf*ACS10.

In addition to the FA species, we also investigated the lipid species most affected by the compounds. Treatment of early trophozoite parasites with MMV665924 or MMV897615 led to a significant decrease in TAGs. Interestingly, the FA moieties most depleted in TAGs (18-carbon length) were also the most affected in the total FA analysis by GC-FID. A role for ACS in TAG formation has been shown in prostate cancer cells where knockdown of ACSL1 resulted in reduced TAG levels^57^. Dephosphorylation of phosphatidic acid yields DAG which can then be converted to TAG through the addition of an acyl-CoA. We observed an increase in phosphatidic acids and DAGs, which could be a direct result from the decrease in TAGs. Phosphatic acid has been shown to play a major role in lipid homeostasis and lipid droplet formation^58^. A reduction in TAGs late in asexual blood stage development could lead to fewer lipid droplets in the parasite, resulting in a lack of stored FAs needed for cellular processes including the generation of membrane precursors for the formation of merozoites during schizogony. Indeed, late-stage parasites treated with any of the three compounds fail to form proper merozoites indicating a defect in membrane formation.

Our work here presents *Pf*ACS10 as a novel target for antimalarials but one that could have potential resistance liabilities. We observed a high degree of genetic variation and divergence within and between *Pf*ACS10 in genomes of *P. falciparum* parasite populations from diverse geographic locations, indicating that this locus may be vulnerable to selection should specific inhibitors be deployed in the future. Indeed, several non-synonymous variants were expected to occur within the predicted substrate (and compound) binding sites of *Pf*ACS10, including the presence of the M300I variant, found at high allele frequency in Malawi. Malawian isolates carrying this variation phenocopied the levels of resistance to MMV665924 seen in ACS10_M300I_S_ and ACS10_M300I_C_ lines. However, to conclusively prove the role of M300I in the resistance to MMV665924 in Malawian isolates, conversion of the M300I mutation to WT in Malawian isolates by CRISPR/CAS9 editing will be needed. *Pf*ACS10 variations have not been associated with any current or prior antimalarial therapies, suggesting that the locus may be under selective pressure due to host-related factors (human or mosquito), such as metabolism. However, very few mutations were observed in *Pf*ACS11 in natural parasite populations. This could indicate that *Pf*ACS11 is not under diversifying selection in the field or that its function is more conserved and might be essential for transmission in the mosquito or in liver stage survival.

The genetic diversity of *Pf*ACS10 and its ability to tolerate mutations could also be used to an advantage. Mutations in *Pf*ACS10 in two parasite lines that have been selected with MMV897615 were either 2.5-fold more sensitive to MMV665924 or 8.5-fold more sensitive to MMV019719 (ACS10_A268V_S_ and ACS10_A268D_S_ respectively). This phenomenon is reminiscent of collateral sensitivity, where resistance to one drug increases the susceptibility to another as has been shown in bacteria (reviewed in^59^) and is studied in cancer treatment^60^. Collateral sensitivity has also been shown in *P. falciparum* for dihydroorotate dehydrogenase^61^ and proteasome inhibitors^9,62^ as well as for dihydrofolate reductase inhibitors in *P. vivax*^63^. Simultaneous or sequential use of distinct *Pf*ACS10 inhibitors might reduce the occurrence of resistance or kill resistant parasites more effectively, making this enzyme a desirable target.

## Methods

### Sources of compounds and parasite lines and culturing

MMV019719, MMV665924, and MMV897615 were freely available as part of the Medicines for Malaria Venture’s (MMV) Malaria Box. Triacsin C was purchased from Sigma-Aldrich (St. Louis, MO, ref. T4540). The 3D7 *P. falciparum* strain was originally obtained through MR4 as part of the BEI Resources Repository, NIAID, NIH: 3D7, MRA-102, deposited by D. J. Carucci. We also used 3D7- IG06 (a fast-growing 3D7 clone)^64^ donated by Daniel Goldberg and Dd2-Polδ^23^ donated by Marcus Lee. The Malawi parasites (CF04.008 10B and CF04.009 6D and 1 G, two different patient isolates that were subcloned after culture adaption) were kindly donated by Danny Milner^26^. Parasites were cultured by standard methods^65^ in RPMI 1640 medium supplemented with 28 mM NaHCO_3_, 25 mM HEPES, 400 μM hypoxanthine, 25 μg/mL gentamicin, and 0.5% AlbuMAX II (Life Technologies, Carlsbad, CA 11021-045). The human erythrocytes were sourced ethically, and their research use was in accord with the terms of approved protocol.

### Generation of allelic replacements with CRISPR/Cas9

Two kinds of plasmids were generated for transfection of 3D7 parental lines. The pDC2-cam-Cas9-U6-hDHFR vector^66^ was used to generate a specific double strand break and a pGEM-3z vector (Promega, Madison, WI, USA) with the homology region containing the single nucleotide polymorphism (SNP) of interest and scrambled guide RNAs (gRNAs) as the repair template. gRNAs were designed using benchling (Benchling, Inc.) and ordered as primers from Integrated DNA Technologies (IDT, Coralville, Iowa, USA) with overhangs compatible with the BbsI overhangs in pDC2-cam-Cas9-U6-hDHFR. The pDC2-cam-Cas9-U6-hDHFR plasmid was digested with BbsI and ligated with the annealed gRNAs. To generate the repair template plasmid, approximately 500 base pairs surrounding the SNP of interest were amplified from gDNA of 3D7 parasites and ligated into pGEM-3z vector using HincII. The SNP of interest and the scrambling of the gRNAs was introduced with the Q5® site-directed mutagenesis kit (New England Biolabs, Ipswich, MA, USA) following manufacturer’s instructions. Sorbitolsynchronized ring-stage parasites were electroporated using a Bio-Rad Gene Pulser (Biorad, Hercules, CA) and conditions of 0.31 kV and 960 µF with a total of 100 μg plasmid template and two pDC2-cam-Cas9-U6-hDHFR plasmids with two different gRNAs in incomplete cytomix using a 0.2-cm cuvette. Resulting transfected parasite lines were cloned by limiting dilution and sequenced by Sanger sequencing to confirm successful gene editing. Sequences of gRNAs and primers used are indicated in Supplementary Table 1.

### Dose response assay phenotyping for *P. falciparum* asexual blood stage

In vitro drug sensitivities of asexual blood stage parasites were determined using a SYBR Green I-(Life Technologies, S7567) based cell proliferation assay^67^. Twelve-point curve dilution series of the test compound were carried out in triplicate on the same day and replicated on at least three different days. The EC_50_ values were calculated using non-linear regression curve fitting in Prism 7 (GraphPad Software Inc., San Diego, CA).

For complementation of minimal FA studies, parasites were resuspended in minimal media that was generated by complementing RPMI-1640 with 0.39% fatty acid-free BSA and oleic and palmitic acid (30 μM each; added from 30 mM ethanol-solved stocks; all from Sigma-Aldrich). Linoleic, cis vaccenic, myristic, palmitoleic and stearic acids were added at 30 μM each to (added from 30 mM ethanol-solved stocks all from Sigma-Aldrich).

### Selection of Dd2-Polδ with MMV897615

The EC_50_ was initially determined to be 32.7 nM in *P. falciparum* Dd2-Polδ^23^. One single-step selection was set up using clonal 1E9 Dd2-Polδ parasites in duplicate at a starting concentration of 3 x EC_50_ (98 nM). Drug pressure was gradually increased when parasites remained healthy at the initial concentration. On day 7 the drug pressure reached its highest (198 nM) and parasites cleared. Shortly after, on day 14, parasites in both flasks recrudesced. These parasites were maintained under the maximum drug dose during culturing.

### Drug susceptibility assays for Dd2-Polδ selected lines

To define the EC_50_ of parasites, ring-stage cultures at 0.2% parasitemia and 1% hematocrit were exposed for 72 h to a range of ten drug concentrations that were 2-fold serially diluted in duplicates along with DMSO controls. Parasite survival was assessed by flow cytometry on an iQue flow cytometer (Sartorius) using SYBR Green and MitoTracker Deep Red FM (Life Technologies) as nuclear stain and vital dyes respectively.

### Whole-Genome sequencing analysis of MMV897615 selected parasites

Whole-genome sequencing for MMV897615 selected clonal parasites was performed using Nextera Flex DNA library kit and multiplexed on a MiSeq flow cell to generate 300 bp paired-end reads. Sequences were aligned to the *Pf*3D7 reference genome (PlasmoDB-48_Pfalciparum3D7; https://plasmodb.org/plasmo/app/downloads/release-48/Pfalciparum3D7/fasta/) using the Burrow-Wheeler Alignment (BWA version 0.7.17). PCR duplicates and unmapped reads were filtered out using Samtools (version 1.13) and Picard MarkDuplicates (GATK version 4.2.2). Base quality scores were recalibrated using GATK BaseRecalibrator (GATK version 4.2.2). GATK HaplotypeCaller (GATK version 4.2.2) was used to identify all possible single nucleotide variants in test parasite lines filtered based on quality scores (variant quality as function of depth QD > 1.5, mapping quality > 40, min base quality score > 18, read depth > 5) to obtain high quality SNPs that were annotated using SnpEff version 4.3t^68^. BIC-Seq version 1.1.2^69^ was used to discover copy number variants (CNVs) against the parental strain using the Bayesian statistical model. SNPs and CNVs were visually inspected and confirmed using Integrative Genome Viewer (IGV). All gene annotations in the analysis were based on PlasmoDB-48_Pfalciparum3D7 (https://plasmodb.org/plasmo/app/downloads/release-48/Pfalciparum3D7/gff/).

### Population genetic analysis

Diversity calculations for genes in Senegal and Malawi were made as previously described^70^. In brief, genome-wide SNP calls were obtained from the Pf3k project (release 5; www.malariagen.net/projects/pf3k). 99 Senegal and 110 Malawi samples were determined to be derived from single-clone infections based on heterozygous call rates (either ≥2% heterozygous calls or ≥4% missing calls) and were retained for downstream analysis. Variants within these samples were masked if they failed any GATK filter, were missing from >25% of samples, or showed high levels of heterozygosity within single infections (>25%). Additionally, 558 genes failed gene-level quality filters (≥20% heterozygous calls or ≥20% missing calls across samples) and were removed from the analysis. Calculations of pairwise nucleotide diversity (π) and Weir and Cockerham’s *F*_*ST*_ estimator were performed the PfalGeneDiversityStats package^71^.

### Structural Modeling of *Pf*ACS10 and *Pf*ACS11

Structural predictions of *Pf*ACS10 and *Pf*ACS11 proteins were obtained from the AlphaFold database^27,72^, AlphaFold DB version 2022-06-01, created with the AlphaFold Monomer v2.0 pipeline). The AlphaFold algorithm incorporates evolutionary, physical and geometric constraints using neural network architectures to predict protein structures. Images were rendered using Pymol 2.5.3.

### Generation of constructs and parasite transfections for translation regulation

To generate the donor vectors for inducible regulation of *Pf*ACS10 (PF3D7_0525100) expression, the right homology regions (RHR) were amplified by PCR using primers listed in Supplementary Table 1, and along with the left homology regions (LHR) and single gRNA fragments synthesized using the BioXP™ 3200 System (SGI-DNA, San Diego, CA), were cloned into the pJazz system-based vector, pSN054^73^. The features of this vector include (i) C-terminal epitope tags V5 and 2x-hemagglutinin (HA) in-frame of the target gene followed by a 10× aptamer array; (ii) a TeTR-DOZI cassette containing the *blasticidin S-deaminase* for selection in the parasites, the reporter gene *Renilla luciferase* (*RLuc*), and the regulatory fusion proteins TetR-DOZI all expressed under the *PfHsp86* promoter and *PfHrp2* terminator; and (iii) a gRNA expression cassette with T7 promoter and terminator. Donor vector generation was carried out via Gibson assembly, and the final construct was sequence verified and further confirmed by restriction digests.

Transfection into parasites was carried out by preloading erythrocytes with the linear vector ACS10_pSN054 as described previously^74^. Briefly, 50 μg of purified plasmid DNA was mixed with human RBC and subjected to 8 square wave electroporation pulses of 365 V for 1 ms each, separated by 0.1 s in a 0.2 cm cuvette. The plasmid DNA preloaded cells were inoculated with NF54 expressing Cas9 and T7 RNA polymerase, maintained in 500 nM anhydrotetracycline (aTc, ref no 37919 Sigma-Aldrich), and drug selection with 2.5 μg/mL of blasticidin (ref no B12150-0.1 RPI Corp, Mt Prospect, IL) was initiated four days after transfection. Emergence of transfected parasites was monitored via Giemsa smears and RLuc measurements. Although transfections with ACS10_pSN054 initially failed, removal of the epitope tags resulted in successful transfections.

### Growth assay

Assessment of parasite proliferation rate over two intra-erythrocytic developmental cycles by titrating the expression of *Pf*ACS10 and YFP was carried out by maintaining the cultures in varying aTc concentrations and using luminescence as a readout of growth. In a 96-well U-bottom BD Falcon™ plate, synchronous ring-stage parasites were set up in triplicate and cultured in the presence (50 and 3 nM) or absence of aTc. Expansion was measured at 0, 72, and 120 h by quantitating luminescence using the Renilla-Glo(R) Luciferase Assay System (ref no E2750, Promega) and the GloMax® Discover Multimode Microplate Reader (Promega). The luminescence values were normalized to chloroquine-treated (200 nM) samples, and results were visualized on a scatter plot using GraphPad Prism (version 8; GraphPad Software).

### *Pf*ACS11 dimerization by western blot

Synchronized ACS11_cKD_ parasites were pelleted 40 h post-invasion and resuspended in 0.03% saponin in 1× PBS at 4 °C for 15 min. Pellets were washed 4 times in 10 volumes of cold PBS with protease inhibitor and resuspended in PBS containing protease inhibitor (ref no 4693159001, Sigma). Half of the sample was transferred to a new tube and incubated with 10μM MMV665924 and MMV019719 each at room temperature for 30 min. Half of each sample was again put in a new tube and mixed with only Laemmli buffer (ref no 1610737, BioRad) for non-reducing conditions or Laemmli buffer containing 2-mercaptoethanol for reducing conditions. Protein samples were run on Mini-PROTEAN® TGX™ Precast Gels (4–20% gradient, BioRad) in either tris-glycine buffer only (non-reducing) or with SDS for reducing conditions. Polyacrylamide gel electrophoresis separated proteins were transferred to a nitrocellulose membrane (Life Technologies, IB3010-31) using the iBlot system (Life Technologies) according to the manufacturer’s instructions and blocked with Intercept® (TBS) Blocking Buffer (LI-COR Biosciences) over night. Membrane-bound proteins were probed with mouse anti-HA (1: 1000; Sigma-Aldrich, H3663) and rabbit-anti H3 (1: 1000 Histone H3 (D1H2) XP® Rabbit mAb, Cell Signaling technology, 4499 S) primary antibody and anti-mouse (IRDye® 680RD goat anti-mouse, 926-68070, LI-COR) and anti-rabbit (IRDye® 800CW Goat anti-Rabbit, 926-32211, LI-COR) secondary antibodies. Protein blots were imaged and analyzed using the LI-COROdyssey CLx Imager System (LI-COR Biosciences) and Image Studio™ Lite (version 5.2.5).

### Immunoprecipitation assays

For co-immunoprecipitation studies, ACS11_cKD_ parasites (250 mL @3% parasitemia and 5% hematocrit) were sorbitol-synchronized, washed and grown either in the presence (500 nM) or absence of aTc, and collected at the schizont-stage (32–42 h post invasion). At collection, erythrocytes were lysed in 0.05% saponin in PBS with EDTA-free protease inhibitor cocktail (cOmplete mini, Sigma), and washed with PBS including protease inhibitors to remove residual erythrocyte material. Parasite pellets were lysed in RIPA buffer (150 mM NaCl, 1% NP-40, 0.5% sodium deoxycholate, 0.1% sodium dodecylsulfate, 50 mM Tris-HCl, pH 7.5, EDTA-free complete mini protease inhibitors (ref no 4693159001, Sigma) for 1 h, sonicated three times at 25% amplitude for 30 s every 3 min, and spun at 12,000 g for 30 min to collect the supernatant as soluble protein. Protein solutions were applied to 40 μL of anti-HA magnetic beads (ref no 88836, Pierce) and incubated for 1 h at room temperature. Beads were washed four times with RIPA buffer, followed by four times with 50 mM ammonium bicarbonate, resuspended in 40 μL ammonium bicarbonate, and submitted for on-bead digestion followed by peptide identification by LC–MS/MS.

### *P. falciparum* lysate preparation for thermal proteome profiling

For TPP experiments, cultures were maintained at a hematocrit of between 1.5– 2.0% with daily or twice daily media changes. Once cultures reached between 8−15% parasitaemia and were late trophozoites, infected erythrocytes were isolated via MACS separation using a SuperMACS II magnet in conjunction with a D column (Miltenyi Biotec). Eluate pellets were resuspended in 10 mL complete media and incubated in drug at 10× EC_50_ or diluent (DMSO) for 1 h at 37 °C in a humidified atmosphere of 1% O_2_, 3% CO_2_ in a balance of N_2_. Drug pressure was maintained throughout the remainder of sample processing. Following centrifugation, parasitised erythrocytes were lysed by incubation in 0.1% (w/v) saponin on ice for 10 min with gentle mixing. The pellet was washed 3× in Wash Buffer (WB; 100 mM potassium acetate, 2.5 mM magnesium acetate, 45 mM HEPES [pH 7.4], 250 mM sucrose, 2 mM dithiothreitol, 15 µM leupeptin) to remove lysed red blood cell material. The pellet was resuspended in one volume of WB supplemented with 0.8% (v/v) *n*-octylglucoside and protease inhibitor (Roche cOmplete EDTA-free protease inhibitor; 1 tablet/ 20 mL) and the parasites lysed by nitrogen cavitation (Parr) (4 °C, 1500 psi, 60 min). The resulting lysate was centrifuged (100,000×*g*, 20 min, 4 °C), the supernatant harvested, and the protein concentration of the lysate determined using the Bio-Rad Protein Assay.

TPP assays were performed as previously described^75^. However, in this instance lysates were exposed to 8 temperatures: 37, 42, 47, 52, 57, 62, 67, and 72 °C.

### Sample processing and fractionation for TPP

Protein digestion was performed as previously described^75^. Samples were then vacuum-dried and resuspended in 100 mM TEAB (100 µL) prior to incubation with their respective Tandem Mass Tag™ (TMT pro) 16-plex reagents (Thermo) for 1 h at room temperature with agitation. Reactions were quenched by the addition of 5% (v/v) hydroxylamine for 15 min, and each set of samples (treated and vehicle) was then pooled and dried overnight. The TMT-labeled samples were dried and desalted as previously described^75^, then kept at −80 °C until further analysis. Sample fractionation was performed as previously described^75^, but with a different gradient adapted to TMT pro-labeled peptides: 2% buffer B to 20% B in 8 min then from 20% B to 40% B in 37 min. The column was washed for 15 min at 100% buffer B and re-equilibrated at 2% buffer B for 20 min.

### LC–MS/MS analysis for TPP

Analysis of peptides was performed on a Orbitrap Eclipse (Thermo Scientific) mass spectrometer coupled to a Dionex Ultimate 3000 RS (Thermo Scientific). Online HPLC was performed as previously described^75^. Orbitrap Eclipse was used in data-dependent mode. A scan cycle comprised MS1 scan (m/z range from 380–1500, with an automatic maximum ion injection time, a resolution of 120,000 and a standard automatic gain control (AGC) target value) followed by sequential dependant MS2 scans (with an isolation window set to 0.7 Da, maximum ion injection time at 50 ms and standard AGC target) and MS3 scans (with a resolution of 50,000, an isolation window set to 0.7 Da, maximum injection time at 120 ms and 400% AGC target). The real-time search feature was active during the analysis.

### Data analysis for TPP

Analysis of the resulting MS data was performed using the software MaxQuant (http://maxquant.org/, version 2.0.3.0). Modifications, digestions, and database search settings were as previously described. Reporter ion MS3 mode was selected using the TMT-16plex labels on N-terminus and lysine. FTMS MS/MS mass tolerance was set to 10 ppm and ITMS MS/MS mass tolerance was 0.5 Da.

TPP experiments were analyzed using the TPP Package available in Bioconductor, as previously described^39,75,76^. Briefly, raw protein abundance, calculated from the normalized reporter ion intensities of all quantified proteins, were normalized to the protein abundance at the lowest temperature for each condition and replica. Melting curves were calculated using a sigmoidal fitting algorithm in the R program of the TPP Package. This fitting was used to determine the melting point (*T*_m_), which is defined as the temperature at which half of the protein was denatured. The melting point differences (Δ*T*_m_) were calculated by subtracting the *T*_m_ values of treated and untreated sample. The sigmoidal melting curves were filtered according to the following criteria: melting curves must reach a relative abundance plateau <0.3 and the correlation coefficient (*R*^2^) must be >0.8. The statistical significance was calculated using a z-test and only proteins with a *p*-value < 0.2 in both technical replicates were considered hits. Hits found in two biological replicates were considered putative targets. Alternatively, we used the NPARC method (non-parametric analysis of response curves), a strategy that compares the experimental data to two models: a null model that assumes that drug has no influence on the protein melting behavior, and an alternative model that assumes that drug affects the melting behavior. Any data-driven adjustments to these models were calculated and assessed for statistical significance using an F-test, generating a *p*-value. All TPP datasets generated with MMV897615 have been deposited to the ProteomeXchange Consortium via the PRIDE partner repository^77^ under the identifier PXD034937.

### Sample preparation for fatty acid composition by gas chromatography-flame ionization detection

To determine whether treatment of parasites with MMV665924 had an influence on the FA composition, highly synchronized 3D7 wildtype or ACS10_M300I_C_ parasites were isolated using a quadroMACS™ magnet with LD columns (130-042-901, Miltenyi Biotec) at around 32 h after invasion. Parasites were then exposed to 10 μM of MMV665924 for 10 h in complete media after which parasites were washed three times with 1× PBS and stored at −80 °C for further extraction.

To test the short-term effect of removal of *Pf*ACS10, parasites with the conditional knockdown lines were synchronized and aTc was washed off at around 20 h post invasion by washing parasites three times in incomplete RPMI for 5 min. Parasites were then either returned to media containing 500 nM aTc (control) or grown in the absence of aTc for 24 h after which they were purified using MACS magnet as described above. Roughly 10^8^ cells were submitted for each sample determined by cell counting on the MACSQuant VYB (Milteni Biotec).

### Gas chromatography-flame ionization detection for FAs

Sample extraction, chromatography, and data analysis were performed at the Nutrition Department GC-FID core facility (Harvard T.H. Chan School of Public Health, Boston, MA). Briefly, total lipids were extracted from erythrocyte into isopropanol and hexane containing 50 mg 2.6-ditert- butyl-p-cresol as an antioxidant^78^. FAs were transmethylated with methanol and sulfuric acid as described^79,80^. After esterification, samples were evaporated, and the FA methyl esters were redissolved in iso-octane. FAs were separated using a Hewlett-Packard Model (now Agilent) GC 6890 FID gas chromatograph with 7673 Autosampler injector (Palo Alto, CA), splitless injection port at 240 °C, and a constant flow hydrogen carrier gas at 1.3 ml/min. 1 μl of sample was injected into a fused silica capillary cis/trans column SP2560, 100 meters × 250 μm internal diameters × 0.20 μm film (Supelco, Belefonte, PA), and run through a temperature program of 90 to 170 °C at 10 °C/min, 170 °C for 5 min, 170 to 175 °C at 5 °C/min, 175 to 185 °C at 2 °C/min, 185 to 190 °C at 1 °C/min, 190 to 210 at 5 °C/min, 210 °C for 5 min, 210 to 250 °C at 5 °C/min, and 250 °C for 10 min. Peak retention times were identified by injecting known standards with purity ranges above 99 percent (NuCheck Prep, Elysium, MN), using Agilent Technologies ChemStation A.08.03 software for analysis. A total of 45 FAs can be identified with this methodology.

### Sample preparation for liquid chromatography mass spectrometry

For lipid profiling, highly synchronized 3D7 wildtype parasites were isolated using a quadroMACS™ magnet with LD columns (130-042-901, Miltenyi Biotec) at around 32 h after invasion. Parasites were then exposed to 10 μM of MMV665924 or 1 μM MMV9897615 for 8 h in complete media after which parasites were washed three times with 1x PBS and cell counts established on a MACSQuant VYB (Milteni Biotec).

Using glass pipettes, parasite pellets were resuspended in 200 μl dH_2_O and then transferred to a glass vial (VWR 66011-550) and homogenized in 2 ml methanol. Four ml of chloroform was then added and the solution vigorously vortexed for 1 min. 1.8 ml of dH_2_O was added and vigorously vortexed for 1 min. Vials were centrifuged for 10 min at about 3000 rcf. The bottom chloroform phase was transferred to a new glass vial and stored at −80 °C.

### Liquid chromatography mass spectrometry

Lipid samples were analyzed at the Harvard Center for Mass Spectrometry. The LC–MS analyses were modified from Miraldi^81^ and were performed on an Orbitrap Exactive plus (Thermo Scientific) in line with an Ultimate 3000 LC (Thermo Scientific). Each sample was analyzed in positive and negative modes, in top 5 automatic data-dependent MSMS mode. Column hardware consisted of a Biobond C4 column (4.6 × 50 mm, 5 μm, Dikma Technologies). Flow rate was set 100 μl min^−1^ for 5 min with 0% mobile phase B (MB), then switch to 400 μl min^−1^ for 50 min, with a linear gradient of MB from 20 to 100%. The column was then washed at 500 μl min^−1^ for 8 min at 100% MB before being reequilibrated for 7 min at 0% MB and 500 μl min^−1^. For positive mode runs, buffers consisted for mobile phase A (MA) of 5 mM ammonium formate, 0.1% formic acid and 5% methanol in water, and for mobile phase B (MB) of 5 mM ammonium formate, 0.1% formic acid, 5% water, 35% methanol in isopropanol. For negative runs, buffers consisted for MA of 0.03% ammonium hydroxide, 5% methanol in water, and for MB of 0.03% ammonium hydroxide, 5% water, 35% methanol in isopropanol. Lipids were identified and quantified using the Lipidsearch© software (version 4.2.27, Mitsui Knowledge Industry, University of Tokyo). Integrations and peak quality were curated manually before exporting and analyzing the data in Microsoft Excel.

### Determination of stage activity of compounds

Highly synchronized 3D7 parasites were cultured in a 24 well plate at 5% hematocrit in 2 ml of complete media for the duration of the experiment. Each well was exposed to drug or DMSO at the highest volume of drug used (1 μM, 5 μM, or 10 μM for MMV665924 and MMV019719, 100 nM, 500 nM, or 1 μM for MMV897615) for 12 h at different times throughout the life cycle (0–12 h, 12–24 h, 24–36 h, 36–48 h) or throughout the whole life cycle (0–48 h). At each 12 h time point, drug-exposed parasites were washed in 10 ml incomplete media (without Albumax II) and resuspended in complete media without any drug and a new well of parasites was exposed to the drug treatment. Half of the media was removed and replenished at each timepoint in all wells to ensure enough nutrients to the parasites. Each well that had been previously exposed was smeared and 5 μl were stained with SYBR Green I for Flow cytometry analysis. A final time point was taken 65 h after starting the experiment.

### Flow cytometry to quantify parasitemia

Parasites were stained in 10× SYBR Green I in 1× PBS for 30 min in the dark at 37 °C. The staining solution was removed, and cells were resuspended in five times the volume of the initial volume of PBS. Flow cytometry data acquisition was performed on a MACSQuant VYB (Milteni Biotec) with a 488 nm laser and a 525 nm filter and analyzed with FlowJo 2. RBCs were gated on the forward light scatter and side scatter and infected RBCs were detected in channel B1. At least 100,000 events were analyzed per sample. An example for the gating strategy is shown in Supplementary Fig. 8.

### Statistical analysis

All experiments were performed at least in three biological replicates, unless otherwise noted. The EC_50_ values for dose response curves were calculated using non-linear regression curve fitting in Prism 7 (GraphPad Software Inc., San Diego, CA). Means were compared using a one-way ANOVA with Dunnett’s post-test when more than three strains were compared to a control line and a two-sided unpaired Student’s *t*-test when only two strains were compared to each other (Prism 7 - 9.5.0. or Excel.16.69.1). No statistical methods were used to predetermine sample size, and the researchers were not blinded to sample identity.

### Reporting summary

Further information on research design is available in the Nature Portfolio Reporting Summary linked to this article.

## Supplementary information

Supplementary Information

Reporting Summary

Description of Additional Supplementary Information

Supplementary Dataset 1

Supplementary Dataset 2

Supplementary Dataset 3

Supplementary Dataset 4

Supplementary Dataset 5

Supplementary Dataset 6

Supplementary Dataset 7

Supplementary Dataset 8

Supplementary Dataset 9

Supplementary Dataset 10

## Data Availability

WGS data that support the findings of this study have been deposited in the European Nucleotide Archive (ENA) at EMBL-EBI under accession number PRJEB57553. All TPP datasets generated with MMV897615 have been deposited to the ProteomeXchange Consortium (https://www.proteomexchange.org) via the PRIDE partner repository (https://www.ebi.ac.uk › pride)^77^ under the identifier PXD034937. All other data supporting the findings of this study are available within the article and its Supplementary Information. PlasmoDB data base served as a reference for gene annotation and expression [https://plasmodb.org]. Genome-wide SNP distribution was obtained from the Pf3k project [release 5; www.malariagen.net/projects/pf3k]. Source data are provided with this paper.

## Source data

Source Data

## Code availability

Code used for the population genetic analysis is freely available for re-use and can be found on GitHub (https://github.com/amearly/Dantzler_et_al_Diversity_Calcs; 10.5281/zenodo.7613429) and zenodo (https://zenodo.org/record/7613429#.Y-GYgezML1K)^71^.
