# Supplementary material for: Potent acyl-CoA synthetase 10 inhibitors kill Plasmodium falciparum by disrupting triglyceride formation
Source: Nat Commun. 2023 Mar 16;14:1455. doi: 10.1038/s41467-023-36921-2 (PMC10020447; doi:10.1038/s41467-023-36921-2)
Supplement: Supplementary file 1 — Supplementary Information [file 41467_2023_36921_MOESM1_ESM.pdf]

Supplementary Information

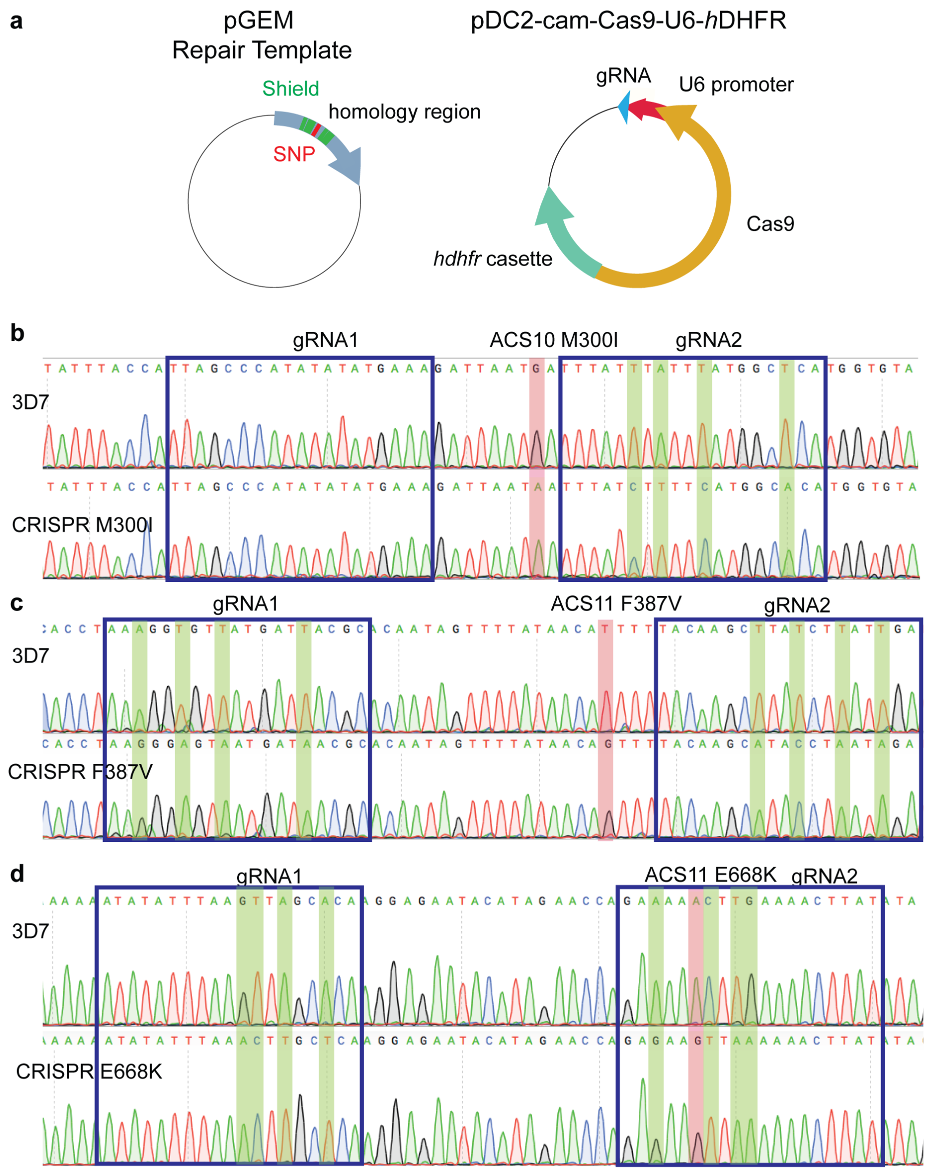

Supplementary Figure 1. Allelic replacement of wildtype sequence by CRISPR editing. a Schematic of the plasmid constructs used for transfection and expression of CRISPR/Cas9 for nucleotide exchange. The plasmid on the left contains the homology region with the single nucleotide polymorphism of interest and shield mutations to inhibit cutting by the Cas9 upon DNA repair. The plasmid on the right contains the Cas9, the gRNA and a selectable marker. **b-d** Electropherograms of the parental line and edited CRISPR lines. The gRNAs used for transfection are boxed. Green shading indicates shield mutations, and red shading shows the nucleotide of interest.

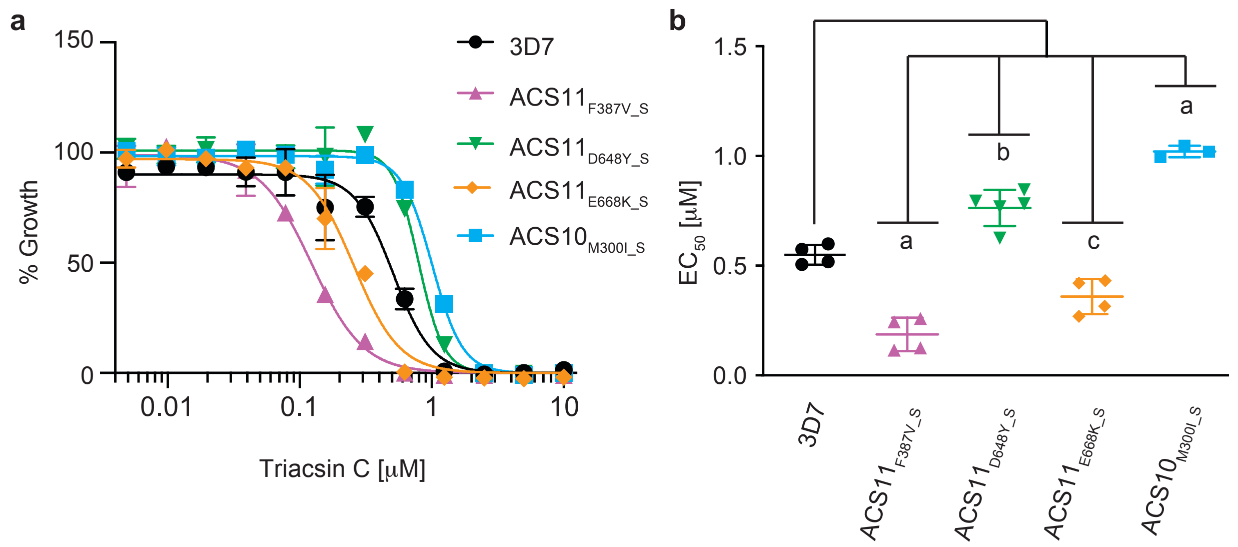

Supplementary Figure 2. Triacsin C treatment of parasites with different mutations in *Pf*ACS10 and *Pf*ACS11results in collateral sensitivity. a Representative dose response assays for Triacsin C for a clonal line of the 3D7 parent (black), and clonal lines from the selections with MMV019719 and MMV665924. Assays were run in triplicate. Shown are the average ± SD and the non-linear regression curve fit for one biological assay run in triplicate. **b** Summary of average EC_{50} values ± SD from all biological replicates performed ($ACS10_{M300I\_S}$ N = 3, 3D7, $ACS11_{F387V\_S}$, $ACS11_{E668K\_S}$ N = 4, and $ACS11_{D648Y\_S}$ N = 5). One-way ANOVA compared to 3D7 with Dunnett's post-test: Significant p values: a < 0.0001, b = 0.0012, and c = 0.0049. Source data are provided as a Source Data file.

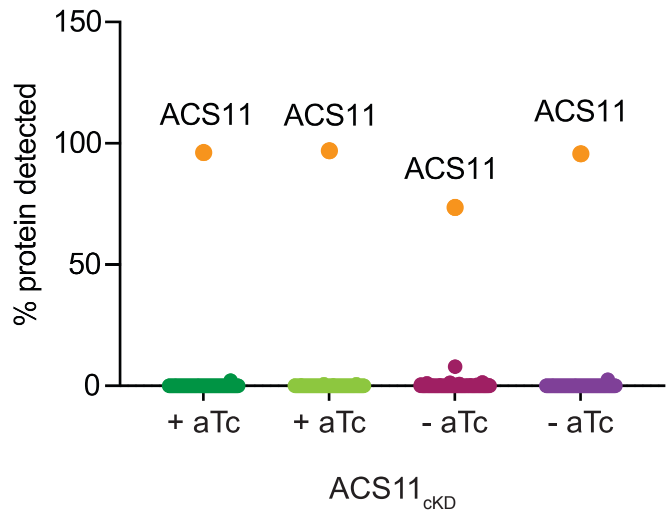

Supplementary Figure 3: *Pf*ACS11 does not interact closely with any other protein. Immunoprecipitation of *Pf*ACS11 followed by LC-MS identified 195 proteins for which two unique peptides were detected in any sample across the dataset. Two biological replicates under high aTc and low aTc conditions were performed. *Pf*ACS11 protein was the dominant protein detected across the two biological replicates under both high aTc and low aTc conditions, with on average >95 % of the peptides detected being attributed to *Pf*ACS11. A list of the proteins identified is given in Supplementary Data 6.

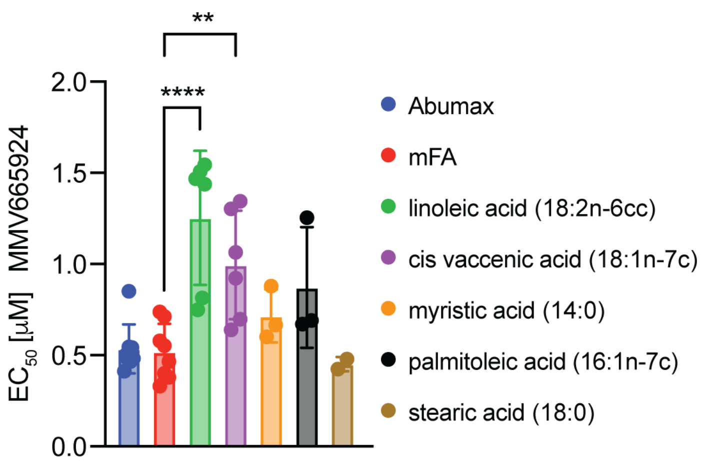

Supplementary Figure 4. Supplementation with linoleic and cis vaccenic acid increases growth under MMV665924 treatment. Mean ± SD EC_{50} values for MMV665924 in Albumax media, minimal fatty acid media or minimal fatty acid media supplemented with one additional fatty acid as indicated in the legend. Assays were run in triplicate and repeated at least twice, except for stearic acid (N = 2). Unpaired ordinary one-way ANOVA with Dunnett's post-test compared to EC_{50} in minimal fatty acid media ** p = 0.005, ****, p < 0.0001. Source data are provided as a Source Data file.

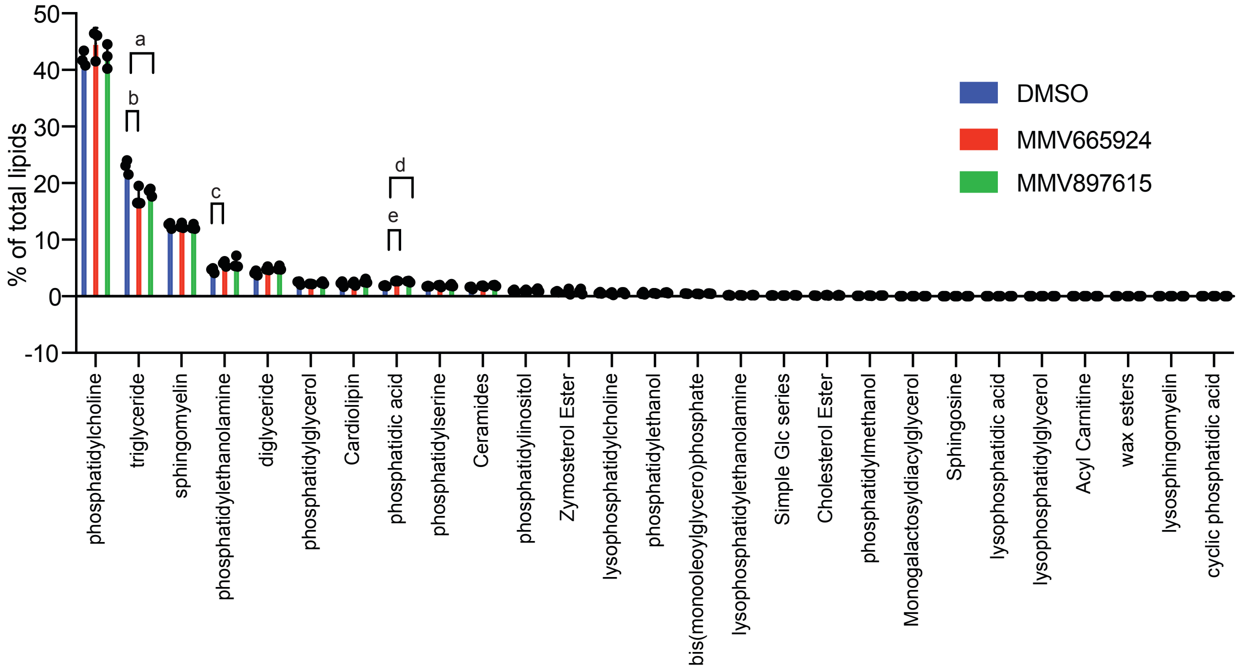

Supplementary Figure 5. Drug treatment inhibits phosphatidic acid to triacylglycerol conversion. Young 3D7 trophozoites were magnet-purified over a MACS column and treated with 10 μM MMV665924 or 1 μM MMV897615 for 8 h. Parasites were extracted and submitted for Liquid Chromatography / Mass Spectrometry (LC/MS); results were normalized to total lipid composition for three biological replicates. Shown are the mean ± SD for the subclasses identified. Tests for significance compared to the DMSO control used two-sided unpaired Student's t-tests; Significant p values: a = 0.006, b = 0.01, c = 0.04, d = 0.001, and e = 0.00009. The complete data are available in Supplementary Data 9.

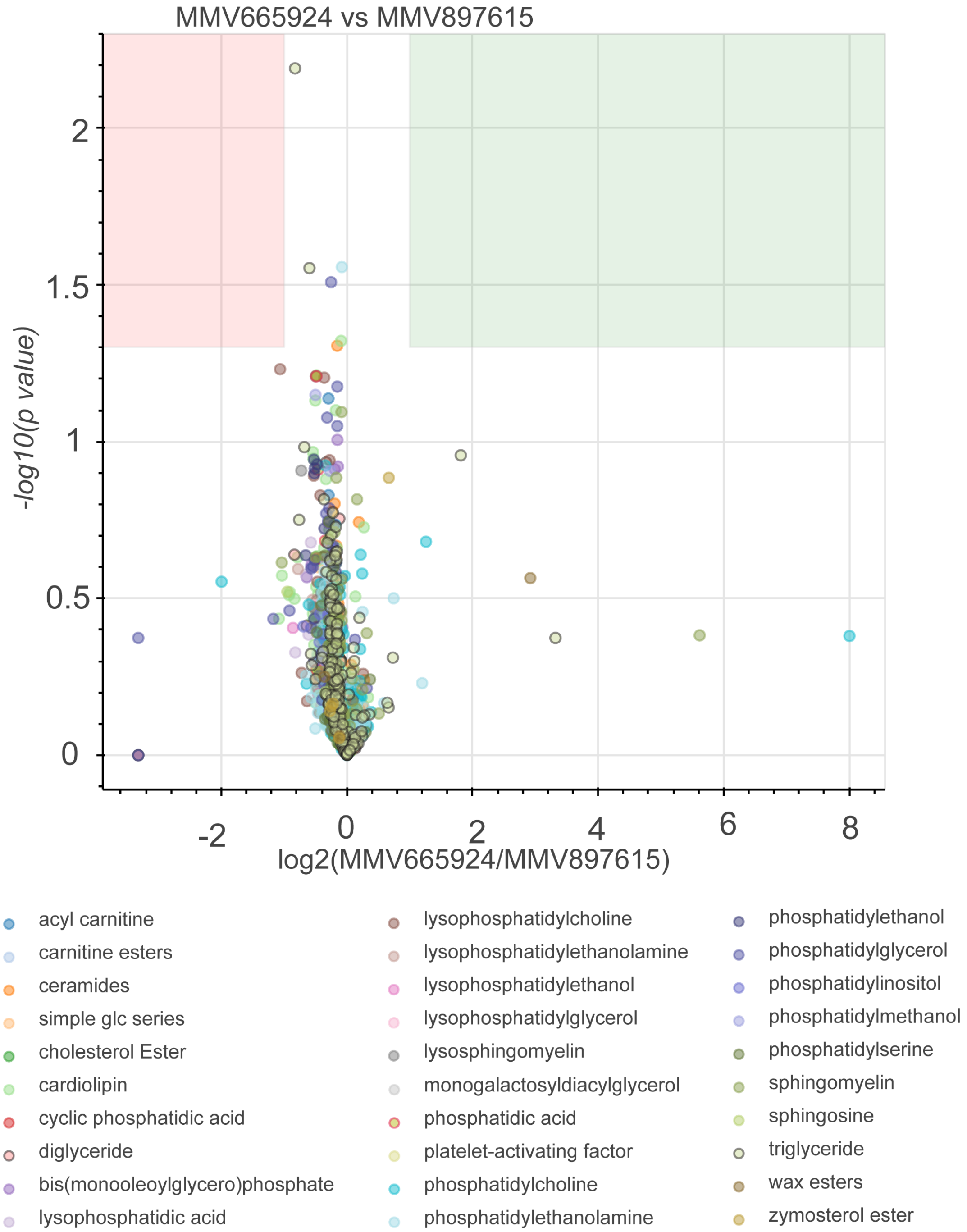

Supplementary Figure 6. MMV655924 and MMV897615 drug treatment elicit similar response in lipid profile of *P. falciparum* parasites. Young 3D7 trophozoites were magnet-purified over a MACS column and treated with 10 μM MMV665924 or 1 μM MMV897615 for 8 h. Parasites were extracted and submitted for Liquid Chromatography / Mass Spectrometry (LC/MS). Results were normalized to total lipid composition for three biological replicates. Volcano plots showing lipid species for MMV665924-treated parasites vs MMV897615-treated parasites. None of the lipid species were more than 2-fold different and had a p value <0.05 (two-sided unpaired Student's t-test). The complete data are available in Supplementary Data 10.

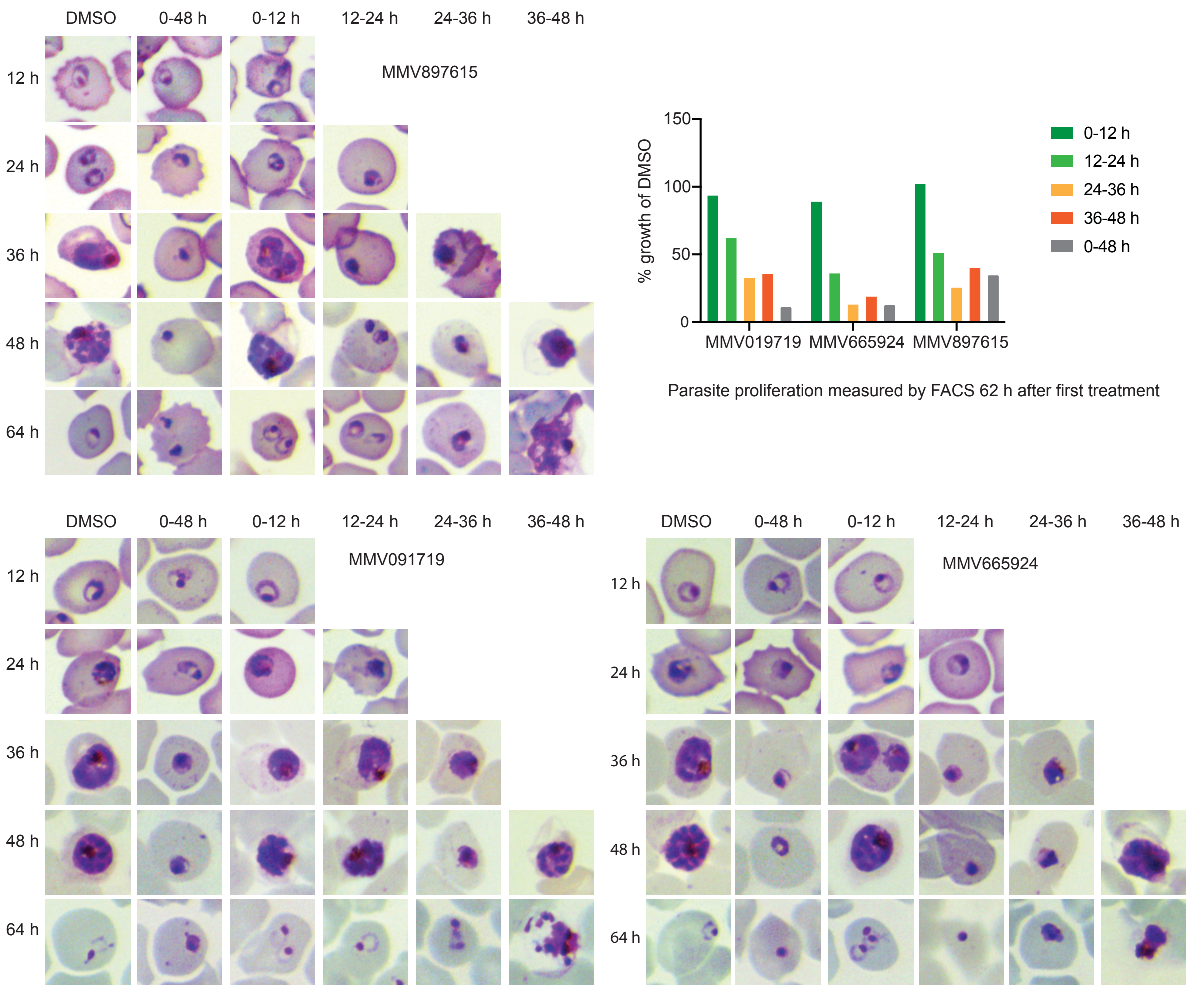

Supplementary Figure 7. MMV019719, MMV665924 and MMV897615 are most active against late-stage parasites. 0-6 h old parasites were exposed for 12 h windows to DMSO, 5 µM MMV019719 or MMV665924, or 500 nM MMV897615. Smears were taken every 12 h after drug exposure and representative images are shown. The parasitemia was measured by flow cytometry in the second lifecycle to determine growth compared to DMSO-treated parasites and is shown in the insets. Gating strategy for flowcytometry is shown in Supplementary Figure 8 and source data are provided as a Source Data file.

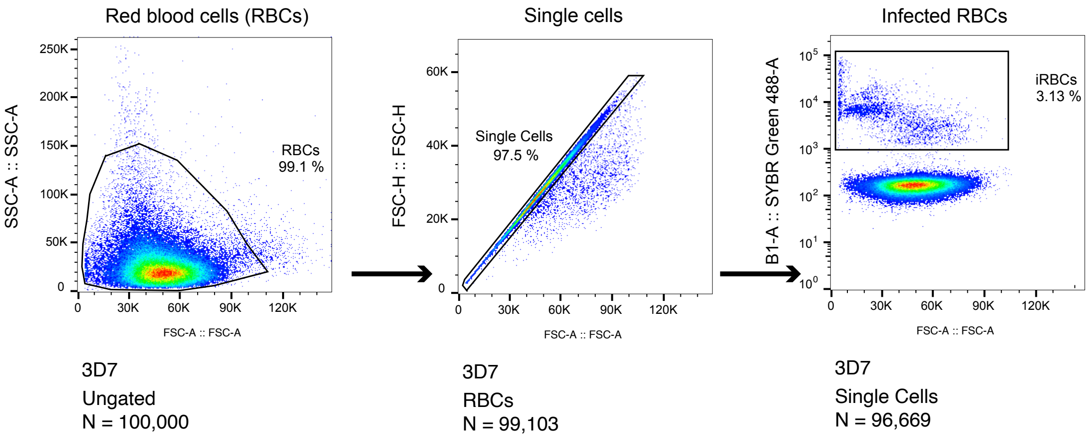

Supplementary Figure 8. Gating strategy for Flow Cytometry analysis of parasitemia. Parasites were stained in 10× SYBR Green I in 1x PBS for 30 minutes in the dark at 37 °C. All red blood cells (RBCs) were first gated on the forward light scatter (FSC) and side scatter (SSC), then single cells were gated on FSC-A vs FSC-H, and finally infected RBCs were detected in channel B1 with laser 488 nm and a 525 nm filter. 100,000 events were analyzed per sample.

Primer name	Sequence
**Homology regions for SNP replacement**	
ACS10 HR_M300I_F	GAGTGTTCAAAATATAATTTGAAAATTATATTATGGG
ACS10 HR_M300I_R	AAAGTAATTTATCCCATAATACATGAGTTGTCG
ACS11 HR_D648Y/E668K_F	AAGTAGGTGTTGGATTTTTACAACATCG
ACS11 HR_D648Y/E668K_R	TTGTTTATTTTCAACAGAGAATGGTGTGG
ACS11 HR_F387V_F	TTTTATGATAAGCTAAAGAAGGAACAAGCAG
ACS11 HR_F387V_R	TCATGAATTTTTTGTAATATTCTTGGTACCG
PF3D7_0525100_pSN054 RHR forward	cagtggtgtacggtacaaacccggaattcgagctcggGTATAAATTTATATTCCGTTAAAAATATATATATACATATCC
PF3D7_0525100_pSN054 RHR reverse	tctggataagacgagagattgggtattagacctagggataacagggtaATGTATATGTCTACACAATTGTTAACAACTTATGGATTC
PF3D7_1238800_pSN054 RHR forward	caaacccggaattcgagctcggattaccctgttatccctaataaaaaaataaaaaaaaaggggtgaaattaaaacagac
PF3D7_1238800_pSN054 RHR reverse	ataaaacatctggataagacgagagattgggtattagaccCATAATTACAACATGTGTTTATATACATATTTATACAATG
**gRNAs**	
ACS10 M300I gRNA1_F	ATTGTTTATTTATTTATGGCTCA
ACS10 M300I gRNA1_R	AAACTGAGCCATAAATAAATAAA
ACS10 M300I gRNA2_F	ATTGTTTCATATATATGGGCTAA
ACS10 M300I gRNA2_R	AAACTTAGCCCATATATATGAAA
ACS11 D648Y/E668K gRNA1_F	ATTGATATATTTAAGTTAGCACA
ACS11 D648Y/E668K gRNA1_R	AAACTGTGCTAACTTAAATATAT
ACS11 D648Y/E668K gRNA2_F	ATTGATAAGTTTTCAAGTTTTTC
ACS11 D648Y/E668K gRNA2_R	AAACGAAAAACTTGAAAACTTAT
ACS11 F387V gRNA1_F	ATTGGCGTAATCATAACACCTTT
ACS11 F387V gRNA1_R	AAACAAAGGTGTTATGATTACGC
ACS11 F387V gRNA2_F	ATTGTACAAGCTTATCTTATTGA
ACS11 F387V gRNA2_R	AAACTCAATAAGATAAGCTTGTA
**site directed mutagenesis primers**	
ACS10_SDM_M300I_F	aataatttatcttttcatggcaCATGGTGTAAAAGTCGGTTATTAC
ACS10_SDM_M300I_R	aatctttcataaatgtgtgcaagtGGTAAATATGATATATGTATATCATTTTC

ACS11_SDM_E668K_R	gaatacatagaaccagagaagttaaaaaacttatatAGTAATAGTATCTATATTGAAAATATTTTTG
ACS11_SDM_E668K_F	tccttgagcaagtttaaatatatttttttgctctatcaATAATTTTTACATATGCATTATTTTCG
ACS11_SDM_F387V_F	ataacagttttacaagcatacctaatagaTGGTAATAGATTAGGTTTAAAAAAATATG
ACS11_SDM_F387V_R	aaaactattgtgcgttatcattactcccttAGGTGTCCCTGAAGAACC
**<u>Homology regions for translation regulation</u>**	
PF3D7_0525100_pSN054 RHR forward	cagtggtgtacggtacaaacccggaattcgagctcgggtataaatttatattccgttaaaaatatatatatacatatcc
PF3D7_0525100_pSN054 RHR reverse	tctggataagacgagagattgggtattagacctagggataacagggtaatgtatatgtctacacaattgttaacaacttatggattc
PF3D7_0525100 ACS10 LHR and re-codonized region	gctcttattggttttcaaacttcattgactgtgccggccggccagaggaccaagtacatgcaacctaggttatttcaaattagaaaaagaaacaaatgaattattagaaaaagatggattcattcgtacaggagatattgccttattaagtccaaatggatccttaaccattatagatagaaaaaagaatatttttaaattagcacaaggagaatatgtagctgtagaaaaagtagaggcatcatataaacaatctttatttattagccaaatttttgtatttggatattcctatgaatctgttctcgtttgtgttatttgtccatcgacagattccatcgatatatggagaacacaaaagaaaatcaaagcaactgatgaagaagtaattaaattaccagaatttaaagcagatgttattaatgatttaacatccatagggaaaaaagacggactcaaaggatttgagcaaattaaggatattcatttcactcttgaggcatttactattgaaaatgatttaatgacacccacaggaaaaattaaaagacatgaagctaagaagagatttaaaaaggaaattgatgagatgtatgaaaagaaactcaaacaggcgatcgcgggtaagcctatccctaaccctctcctcggtctcg
PF3D7_0525100 ACS10 sgRNA	ataacttcgtatagcatacattatacgaagttatggcgcgcccgtaactataacggtcctaaggtagcgaataatacgactcactatagggttgaagcaatagaacataagttttagagctagaaatagcaagttaaaataaggctagtccgttatcaacttgaaaaagtggcaccgagtcggtgctagcataaccccttggggcctctaaacgggtcttgagggtttttttgcggtctcagtggtgtacggtacaaacccggaattcgagctcgg

Supplementary Table 1. List with primers used to generate allelic replacements and conditional knockdown vectors
