# Supplementary material for: Potent acyl-CoA synthetase 10 inhibitors kill Plasmodium falciparum by disrupting triglyceride formation
Source: Nat Commun. 2023 Mar 16;14:1455. doi: 10.1038/s41467-023-36921-2 (PMC10020447; doi:10.1038/s41467-023-36921-2)
Supplement: Supplementary file 2 — Reporting Summary [file 41467_2023_36921_MOESM2_ESM.pdf]

nature research

Corresponding author(s): Dyann Wirth

Last updated by author(s): Feb 7, 2023

Reporting Summary

Nature Research wishes to improve the reproducibility of the work that we publish. This form provides structure for consistency and transparency in reporting. For further information on Nature Research policies, see our Editorial Policies and the Editorial Policy Checklist.

Statistics

For all statistical analyses, confirm that the following items are present in the figure legend, table legend, main text, or Methods section.

n/a	Confirmed	
☐	☒	The exact sample size (n) for each experimental group/condition, given as a discrete number and unit of measurement
☐	☒	A statement on whether measurements were taken from distinct samples or whether the same sample was measured repeatedly
☐	☒	The statistical test(s) used AND whether they are one- or two-sided<br>*Only common tests should be described solely by name; describe more complex techniques in the Methods section.*
☒	☐	A description of all covariates tested
☒	☐	A description of any assumptions or corrections, such as tests of normality and adjustment for multiple comparisons
☐	☒	A full description of the statistical parameters including central tendency (e.g. means) or other basic estimates (e.g. regression coefficient) AND variation (e.g. standard deviation) or associated estimates of uncertainty (e.g. confidence intervals)
☐	☒	For null hypothesis testing, the test statistic (e.g. F, t, r) with confidence intervals, effect sizes, degrees of freedom and P value noted<br>*Give P values as exact values whenever suitable.*
☒	☐	For Bayesian analysis, information on the choice of priors and Markov chain Monte Carlo settings
☒	☐	For hierarchical and complex designs, identification of the appropriate level for tests and full reporting of outcomes
☐	☒	Estimates of effect sizes (e.g. Cohen's d, Pearson's r), indicating how they were calculated

Our web collection on statistics for biologists contains articles on many of the points above.

Software and code

Policy information about availability of computer code

Data collection	no software was used to collect data
Data analysis	Microsoft Excel (version16.69), GraphPad Software Inc (version 9.5.0) , Benchling, Inc. [benchling.com], WGS analysis: Burrow-Wheeler Alignment (BWA version 0.7.17), Samtools (version1.13) and Picard MarkDuplicates (GATK version 4.2.2), GATK BaseRecalibrator (GATK version 4.2.2), GATK HaplotypeCaller (GATK version 4.2.2), SnpEff version 4.3t, BIC-Seq version 1.1.2, Integrative Genome Viewer (version 2.15.4), AlphaFold DB (version 2022-06-01), Pymol 2.5.3., LI- COROdyssey CLx Imager System (LI-COR Biosciences), Image Studio™ Lite (version 5.2.5), MaxQuant (http:// maxquant.org/, version 2.0.3.0), Agilent Technologies ChemStation A.08.03, Lipidsearch© software(version 4.2.27, Mitsui Knowledge Industry, University of Tokyo), FlowJo 2(version 10.5.2), TPP Package available in Bioconductor, R program of the TPP Package

For manuscripts utilizing custom algorithms or software that are central to the research but not yet described in published literature, software must be made available to editors and reviewers. We strongly encourage code deposition in a community repository (e.g. GitHub). See the Nature Research guidelines for submitting code & software for further information.

Data

Policy information about availability of data

All manuscripts must include a data availability statement. This statement should provide the following information, where applicable:

- Accession codes, unique identifiers, or web links for publicly available datasets
- A list of figures that have associated raw data
- A description of any restrictions on data availability

WGS data that support the findings of this study have been deposited in the European Nucleotide Archive (ENA) at EMBL-EBI under accession number PRJEB57553 (https://www.ebi.ac.uk/ena/browser/view/PRJEB57553). All TPP datasets generated with MMV897615 have been deposited to the ProteomeXchange Consortium

(https://www.proteomexchange.org) via the PRIDE partner repository (https://www.ebi.ac.uk › pride)[79] under the identifier PXD034937. All other data supporting the findings of this study are available within the article and its Supplementary Information. Source data are provided with this paper. PlasmoDB data base served as a reference for gene annotation and expression [https://plasmodb.org]. Genome-wide SNP distribution was obtained from the Pf3k project [release 5; www.malariagen.net/projects/pf3k].

Field-specific reporting

Please select the one below that is the best fit for your research. If you are not sure, read the appropriate sections before making your selection.

- [x] Life sciences
- [ ] Behavioural & social sciences
- [ ] Ecological, evolutionary & environmental sciences

For a reference copy of the document with all sections, see nature.com/documents/nr-reporting-summary-flat.pdf

Life sciences study design

All studies must disclose on these points even when the disclosure is negative.

Sample size	Sample size was not determined prior to experiments
Data exclusions	One data set from the four biological data sets for M300I under 10uM treatment was omitted as the total fatty acids amount extracted from the sample was below the cut off considered acceptable for the experiment.
Replication	In general all experiments were performed in at least 3 biological replicates, drug assays were also run in technical triplicates, and reproducibility was good
Randomization	samples were not randomized
Blinding	investigators were not blinded

Reporting for specific materials, systems and methods

We require information from authors about some types of materials, experimental systems and methods used in many studies. Here, indicate whether each material, system or method listed is relevant to your study. If you are not sure if a list item applies to your research, read the appropriate section before selecting a response.

Materials & experimental systems

n/a	Involved in the study	
[ ]	[x]	Antibodies
[ ]	[x]	Eukaryotic cell lines
[x]	[ ]	Palaeontology and archaeology
[x]	[ ]	Animals and other organisms
[x]	[ ]	Human research participants
[x]	[ ]	Clinical data
[x]	[ ]	Dual use research of concern

Methods

n/a	Involved in the study	
[x]	[ ]	ChIP-seq
[ ]	[x]	Flow cytometry
[x]	[ ]	MRI-based neuroimaging

Antibodies

Antibodies used	mouse anti-HA (Sigma-Aldrich, H3663), rabbit-anti H3(D1H2) XP® Rabbit mAb, Cell Signaling technology, 4499S), anti-mouse (IRDye® 680RD goat anti-mouse ,926-68070, LI-COR) and anti-rabbit (IRDye® 800CW Goat anti-Rabbit ,926-32211, LI-COR), anti-HA magnetic beads (ref no 88836, Pierce)
Validation	mouse anti-HA: https://www.sigmaaldrich.com/US/en/product/sigma/h3663<br>rabbit-anti H3: https://www.cellsignal.com/products/primary-antibodies/histone-h3-d1h2-xp-rabbit-mab/4499<br>anti-mouse: https://www.licor.com/bio/reagents/irdye-680rd-goat-anti-mouse-igg-secondary-antibody?utm_source=google&utm_medium=adwords&utm_content=reagent-webpage&utm_campaign=reagents&gclid=Cj0KCQiAkMGcBhCSARIsAIW6d0B6NgLjzLHUun0bbw5xJIDuLdVU740zsQV9oB3JspWhHvvO6F6guBAaAqSMEALw_wcB<br>anti-rabbit: https://www.licor.com/bio/reagents/irdye-800cw-goat-anti-rabbit-igg-secondary-antibody?utm_source=google&utm_medium=adwords&utm_content=reagent-webpage&utm_campaign=reagents&gclid=Cj0KCQiAkMGcBhCSARIsAIW6d0AH-Q6pHgftUHA44eHy5KQ472LRtefKjYffZQpISVEnAwLPzU291ZEaAjKQEALw_wcB<br>anti-HA magnetic beads: https://www.thermofisher.com/order/catalog/product/88836

Eukaryotic cell lines

Policy information about cell lines

Cell line source(s)	Plasmodium falciparum 3D7 MRA-102 from BEI Resources Repository, NIAID, NIH, Dd2-Poldelta was obtained from Marcus Lee, CF04.008 and CF04.009 were donated by Danny Milner, 3D7 IG06 from Daniel Goldberg
Authentication	Genetically manipulated P. falciparum lines were were genotyped by whole genome sequencing.
Mycoplasma contamination	Cell lines were not tested for mycoplasma contamination
Commonly misidentified lines (See ICLAC register)	no commonly misidentified lines were used in this study

Flow Cytometry

Plots

Confirm that:

- [x] The axis labels state the marker and fluorochrome used (e.g. CD4-FITC).
- [x] The axis scales are clearly visible. Include numbers along axes only for bottom left plot of group (a 'group' is an analysis of identical markers).
- [x] All plots are contour plots with outliers or pseudocolor plots.
- [x] A numerical value for number of cells or percentage (with statistics) is provided.

Methodology

Sample preparation	Parasites were stained in 10x SYBR Green I -(Life Technologies, S7567) in 1x PBS for 30 minutes in the dark at 37 °C. The staining solution was removed, and cells were resuspended in five times the volume of the initial volume of PBS.
Instrument	MACSQuant VYB (Milteni Biotec) with a 488 nm laser and a 525 nm filter
Software	FlowJo 10.5.2
Cell population abundance	100,000 red blood cells were detected in total, parasitemia was generally above 1 % (1000 infected cells)
Gating strategy	Red blood cells (RBCs) were gated on the forward light scatter (FSC-A) and side scatter (SSC-A), single cells were gated on FSC-A vs FSC-H, and infected RBCs were detected in channel B1 (AlexaFluor488-A).

- [x] Tick this box to confirm that a figure exemplifying the gating strategy is provided in the Supplementary Information.
