# Supplementary material for: Potent acyl-CoA synthetase 10 inhibitors kill Plasmodium falciparum by disrupting triglyceride formation
Source: Nat Commun. 2023 Mar 16;14:1455. doi: 10.1038/s41467-023-36921-2 (PMC10020447; doi:10.1038/s41467-023-36921-2)
Supplement: Supplementary file 3 — Description of Additional Supplementary Information [file 41467_2023_36921_MOESM3_ESM.docx]

**Description of supplementary information files**

**File name:** Supplementary Data 1

**Description**: All EC_50_ values and statistics for MMV019719, MMV665924, and MMV897615 drug assays.

**File name:** Supplementary Data 2

**Description**: All EC_50_ values and statistics for atovaquone, amodiaquine, mefloquine, quinine and triacsin C.

**File name:** Supplementary Data 3

**Description**: List of SNPs and CNVs identified in whole-genome sequencing of MMV897615 selected clones.

**File name:** Supplementary Data 4

**Description**: Mutations reported in Pf3K data base for *Pf*ACS10 and *Pf*ACS11 with MFA > 0.01.

**File name:** Supplementary Data 5

**Description**: List of all hits identified by thermal protein profiling.

**File name:** Supplementary Data 6

**Description**: List of all peptides detected by *PfA*CS11-HA immunoprecipitation.

**File name:** Supplementary Data 7

**Description**: List and statistics of all fatty acid species detected by GC-FID for *Pf*ACS10_cKd_ line +/- aTc and RBCs.

**File name:** Supplementary Data 8

**Description**: List and statistics of all fatty acid species detected by GC-FID for 3d7 and ACS10_M300I_C_ ± MMV6659245.

**File name:** Supplementary Data 9

**Description**: List and statistics of average percentage of lipid subspecies detected by LC/MS for control or MMV665924- or MMV897615-treated parasites.

**File name:** Supplementary Data 10

**Description**: List and statistics of all lipid species detected by LC/MS for control or MMV665924- or MMV897615-treated parasites.
